# Aroma Profile of Merlot Red Wine Stored in Stainless-Steel Tanks and Wooden Barrels with Different Toasting Methods

**DOI:** 10.3390/foods13010045

**Published:** 2023-12-21

**Authors:** Anita Pichler, Ivana Ivić, Josip Mesić, Mato Drenjančević, Toni Kujundžić, Tanja Marković, Mirela Kopjar

**Affiliations:** 1 Faculty of Food Technology Osijek, Josip Juraj Strossmayer University of Osijek, F. Kuhača 18, 31000 Osijek, Croatia; anita.pichler@ptfos.hr (A.P.); mirela.kopjar@ptfos.hr (M.K.); 2 Faculty of Tourism and Rural Development, Josip Juraj Strossmayer University of Osijek, Vukovarska 17, 34000 Požega, Croatia; jmesic@ftrr.hr; 3 Faculty of Agrobiotechnical Sciences Osijek, Josip Juraj Strossmayer University, V. Preloga 1, 31000 Osijek, Croatia; mato.drenjancevic@fazos.hr (M.D.); toni.kujundzic@fazos.hr (T.K.); 4 Teaching Institute of Public Health for the Osijek-Baranja County, Franje Krežme 1, 31000 Osijek, Croatia; tanja.markovic.zzjz@gmail.com

**Keywords:** stainless steel tank, wooden oak barrel, toasting, wine aroma, storage

## Abstract

Stainless-steel tanks and wooden barrels are the most common wine ageing and storage vessels. Wooden barrels are often toasted to improve their chemical composition and influence on wine. The aim of this study was to investigate the changes in Merlot red wine aroma from the 2020 and 2021 vintages during 12-month storage (with sampling every 3 months) in a stainless-steel tank (SST), Excellence oak barrels with medium (EMT), medium plus (EMT+) and medium long (EMLT) toasting and a Premium oak barrel with medium toasting (PMT). The results showed that even slight differences in the time and temperature of medium toasting influenced the extraction of aroma compounds from wood to wine. The changes in individual aroma compounds depended on the vessel type, toasting level, initial wine composition and storage time. An increase in the total concentration of compounds with smoky, spicy and woody notes occurred in both wine vintages stored in wooden barrels, especially during longer storage. In samples from SST, floral, fruity and herbal aromas were more pronounced, according to the gas chromatography and sensory evaluators. Sensory evaluators rated the samples according to the 100-point test, and after 12 months of storage, 2020 and 2021 vintage Merlot stored in PMT obtained the highest points.

## 1. Introduction

Wine production is a complex process that includes numerous different procedures, from vineyard practices to the vinification process and wine ageing. Each stage is equally important and affects the chemical composition, quality and aroma of the final product. Wine aroma is a complex property that originates primarily from grapes, and then it is changed and developed during fermentation (secondary aroma) and finally during ageing and maturation of wine (tertiary aroma) [1]. There are various factors that affect the aroma profile of must and later wine, like climate conditions (temperature, humidity, precipitation, etc.), soil characteristics and position, conditions during harvest, grape crushing and pressing, alcoholic and malolactic fermentation conditions, maturation, ageing and storage conditions (temperature, time and vessel type) [2,3,4].

Today’s most common wine fermentation or ageing vessel is the stainless-steel tank due to its durability, easy maintenance and manageability. Stainless steel is chemically inert and it does not react with wine, but rather only preserves its original flavour and aroma resulting in a homogenous wine [5]. On the other hand, the wooden barrel is more complex, and its influence on wine depends on various factors, like the type of wood, barrel volume, age and number of uses, toasting level, etc. It is very important to choose the right type of wood for barrel production because the wood should be thermoplastic (bends with heating), without any defects that could cause leakage, and should not contribute any undesirable aroma compounds. Today, wooden barrels are made out of white (*Quercus alba*) or red oak (*Quercus rubra*), chestnut oak (*Quercus montana*), redwood (*Sequoioideae*), sugar maple (*Acer saccharum*), mulberry (*Morus alba*), black cherry (*Prunus serotina*) and many others that satisfy physical and structural characteristics [6]. The most common wood used for barrel production is oak due to its properties (porosity, hardness, aroma contribution and mechanical properties) and geographical availability [7].

The porosity of a wooden barrel allows the microoxygenation of wine, which improves the wine aroma and enhances the interactions between wine and wood. Those interactions result in the transfer of ellagitannins and other compounds into wine, resulting in the polymerisation, agglomeration, condensation or stabilisation of new aroma compounds [8]. However, the porosity of wood also causes wine losses due to the evaporation of wine through the wood and the impregnation of wine into the dry wood. It results in a headspace at the top of the barrel that needs to be filled with additional wine or inert gas. This means that the wooden barrel is an interactive vessel and it is necessary to regularly monitor the wine in it [6].

Besides wood type, wooden barrels can differ in volume and size. Larger barrels have a lower wood surface/wine volume ratio than smaller barrels, resulting in lower compound exchange and interaction. Barrique barrels are used very often and traditionally hold 225 litres [9].

Toasting represents a controlled process of indirect heating of the inside of the wooden barrel that transforms its raw wood aromas into spice, smoke and vanilla aromas and eliminates the wood tannins. The barrel is placed on an iron grill that is ignited at a closely monitored temperature and time. The increase in temperature and toasting time results in the formation of new chemical compounds, but if the temperature is too high or the toasting time is too long, the thermodegradation of desirable compounds could occur [9,10]. There are several toasting levels that are obtained at different temperatures and times, depending on the manufacturer, type of wood and barrel size. Light toasting (LT) is usually conducted at a temperature range of 120 to 180 °C for approximately 5 min. This toasting level is suitable when minimal wine aroma changes and high tannin content are necessary. Medium toasting (MT; 180–190 °C, up to 5 min) results in more complex and stronger vanilla aromas, suitable for full-flavoured wines. Medium plus toasting (MT+) results in more intense aroma compounds than MT, with vanilla, spice and brown sugar notes, due to slightly higher temperatures (190–210 °C, up to 5 min). Medium long toasting (MLT), suitable for highly concentrated red wines and ageing on lees, is conducted at slightly lower temperatures than MT but for 10 min or longer. Heavy toasting (HT) results in the degradation of most oak chemical compounds and tannins due to higher temperatures (up to 230 °C, for 5 to 15 min). It contributes to the deep smoky and coffee aroma of wine [11,12,13].

Many previous studies have been conducted in order to investigate the influence of different ageing vessels, especially toasted wooden barrels, on wine aroma [8,10,14,15,16,17,18] and phenolic, mineral and chemical compositions [19,20,21]. Several studies investigated the use of different types of wooden chips that will imitate the wine’s contact with the wooden barrel [22,23,24,25,26]. However, there is room for further research because there can be numerous variations of wine ageing vessels, different types of wood, toasting levels, different wine varieties, ageing time and storage conditions.

The aim of this study was to investigate the influence of different vessel types (a stainless-steel tank and wooden barrels with medium, medium plus and medium long toasting) on the aroma profile and sensory properties of Merlot red wine during 12 months of ageing (with sampling every 3 months). Merlot red grapes are one of the most widely planted grapes and the most popular wine variety in the world. It has dark blue to black-coloured loose bunches of large berries. It originates from Bordeaux, France, but it can be cultivated in various regions, preferring cold and well-drained soil. The wine produced from this grape variety is usually full-bodied, has a dark red or purple colour, velvety tannins, high phenolic content and herbal and fruity aromas with plum, coffee and dark chocolate notes. Merlot red wine matures faster than Cabernet Sauvignon, but it can also mature and develop for decades, depending on the production method [27]. In this study, two vintages (2020 and 2021) of Merlot wines were used to investigate the repeatability of the vessel influence during storage. Obtained samples were analysed on a gas chromatograph equipped with a mass spectrometer, and expert evaluators conducted the sensory evaluation.

## 2. Materials and Methods

### 2.1. Materials

In this study, the following chemicals were used to determine aroma compounds: myrtenol standard (Sigma-Aldrich, St. Lois, MO, USA) and sodium chloride (Kemika, Zagreb, Croatia).

### 2.2. Barrel Production

The barrels were produced in the Auric Barrels cooperage (Našice, Croatia) from their own wood, using two types of oak wood: sessile oak (*Quercus petraea* L.) and pedunculated oak (*Quercus robur* L.), in a ratio 70:30, respectively. The average age of the oaks used for barrel production ranges from 120 to 140 years. Regarding the air-drying length of the barrel’s staves and the density of the grains, there is a difference between Excellence and Premium barrels. The Excellence barrels have 3 to 5 grains/cm and Premium barrels have 5 to 7 grains/cm. Both types of barrels were produced from staves that were air-dried for 24 to 36 months.

For this study, 4 kinds of barrels with different toasting methods and characteristics were ordered from Auric Barrels cooperage: one Excellence and one Premium barrel with medium toasting, and Excellence barrels with medium plus and medium long toasting. Medium toasting was conducted for 60 min, raising the temperature from 100 °C to 190 °C. Medium plus toasting also lasted 60 min, but the initial temperature was 110 °C and it was increased to 205 °C. Medium-long toasting was conducted at the temperature range of 120–210 °C for 65 min. Two sets of the same barrel types were purchased for both wines, vintage 2020 and 2021.

### 2.3. Wine Production

The harvest of Merlot grapes took place on 11th November 2020 and 1st November 2021 in the Kutjevo vineyard, according to the grape ripeness. After grape mashing and crushing, the obtained mash (must with pulp) was placed in containers for maceration and fermentation. For both vintages, the same vinification conditions were used. Maceration was conducted in stainless-steel vertical Vinimatic for 12 days, where the mash was immersed twice a day. After maceration, the mash was pressed and transferred to a stainless-steel tank. Fermentation was conducted with *Saccharomyces* yeasts, Siha Finesse red, and the fermentation temperature was in the range from 23 to 25 °C. The fermentation and complete vinification process was finished at the beginning of March 2020 (Me20) and May 2021 (Me21). At that point, a sample of finished wine was taken, and the rest was stored in different ageing vessels: a stainless-steel tank (SST), Excellence oak wooden barrels with medium (EMT), medium plus (EMT+), medium-long toasting (EMLT) and a Premium oak wooden barrel with medium toasting (PMT). Samples were taken from each vessel every 3 months for 1 year. Sampling and analyses of all wines were conducted in triplicates. The same procedure was repeated with vintage 2020 and 2021. The differences between the two vintages were climate conditions, like air temperature, sunshine hours and precipitation as presented in Table 1.

### 2.4. Gas Chromatography

Aroma compounds in obtained samples were determined by a gas chromatograph (Agilent 7890B) equipped with a mass spectrometer (Agilent 5977A) and autosampler PAL RSI 120 (Agilent Technologies, Santa Clara, CA, USA). Sampling was carried out by the solid-phase microextraction (SPME) method with the following conditions: sample volume was 5 mL with 1 g of NaCl and 5 µL of myrtenol (0.5 mg/L) as internal standard; SPME fibre filling was a polydimethylsiloxane/divinylbenzene sorbent (PDMS/DVB), 65/10, violet (Agilent Technologies, Santa Clara, CA, USA); sample extraction time with SPME fibre was 45 min at 40 °C in agitator, followed by 7 min of desorption at 250 °C in GC injection port. The gas chromatograph and mass spectrometer (GC/MS) conditions were the same as described in our previous articles [1,28]. Samples were analysed in triplicates, compound identification was based on their mass spectra, retention time and index (calculated according to the C7–C30 saturated alkanes standard retention time), NIST (National Institute of Standards and Technology, Gaithersburg, MD, USA) and Wiley mass spectral database.

### 2.5. Sensory Evaluation of Wine

The sensory evaluation of samples was performed by a panel of certified sensory evaluators who were trained as part of the “Uncorking rural heritage” project. The training was conducted according to the procedure from the Norwegian Institute for Organoleptics (NOFIMA). There were 3 men and 2 women in an age range of 30 to 50 years with more than 5 years of experience in sensory evaluation. The 100-point test of the International Organisation of Vine and Wine (OIV) was used. The results were obtained by eliminating the highest and lowest values and calculating the arithmetic mean values of the three remaining datasets [29]. A descriptive analysis of wine was also conducted, where evaluators ranked from 0 to 10 the intensity of specific aromatic notes that are characteristic of Merlot wine (plum, black cherry, raspberry, berry fruits, blackberry, blueberry, plum jam, dry fig, chocolate, cedar wood, sawdust, cloves, vanilla, caramel, coffee, hazelnuts, spices, fruits and herbal).

### 2.6. Statistical Calculation

For each sample, the average value and standard deviation were calculated. The STATISTICA 13.1 (StatSoft, Tulsa, OK, USA) software program was used for the analysis of variance (ANOVA), Fisher’s least significant difference (LSD) test with *p* < 0.05 and principal component analysis (PCA).

## 3. Results

**Figure 1:**
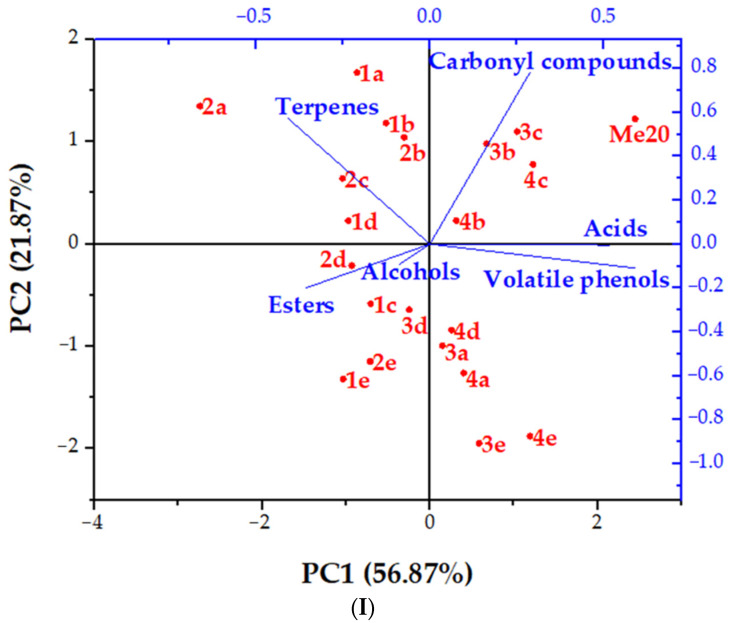

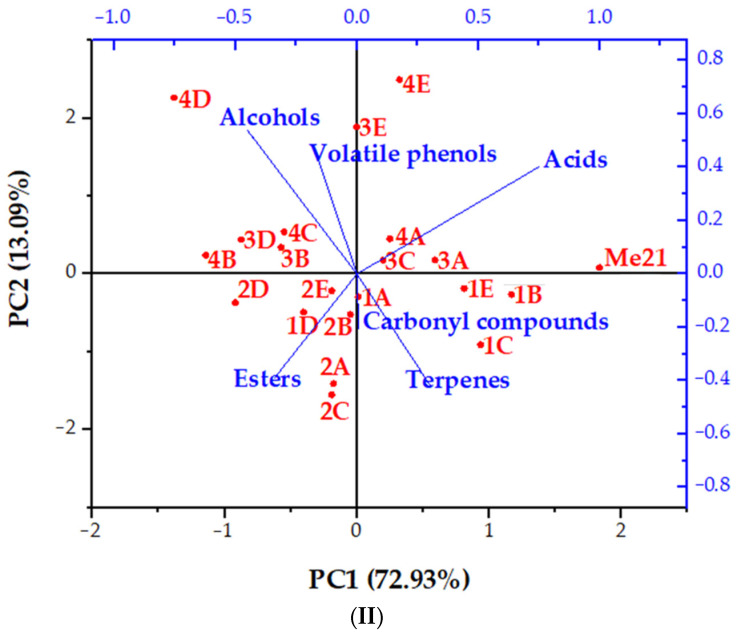
Principal component analysis (PCA) biplot of aroma compounds in vintage 2020 (**I**) and vintage 2021 (**II**) Merlot red wine and samples obtained during 12-month storage in different vessels. Abbreviations: (**I**) Me20—2020 vintage Merlot sample prior storage; a—stainless-steel tank; b—wooden barrel with excellent medium toasting; c—wooden barrel with excellent medium plus toasting; d—wooden barrel with excellent medium long toasting; e—wooden barrel with premium medium toasting; 1—sampling atin June 2021; 2—sampling atin September 2021; 3—sampling atin December 2021; 4—sampling atin March 2022. (**II**) Me21—2021 vintage Merlot sample prior storage; A—stainless-steel tank; B—wooden barrel with excellent medium toasting; C—wooden barrel with excellent medium plus toasting; D—wooden barrel with excellent medium long toasting; E—wooden barrel with premium medium toasting; 1—sampling in August 2022; 2—sampling in November 2022; 3—sampling in February 2023; 4—sampling in May 2023. Red colour–samples; blue colour–groups of volatiles.

**Figure 2:**
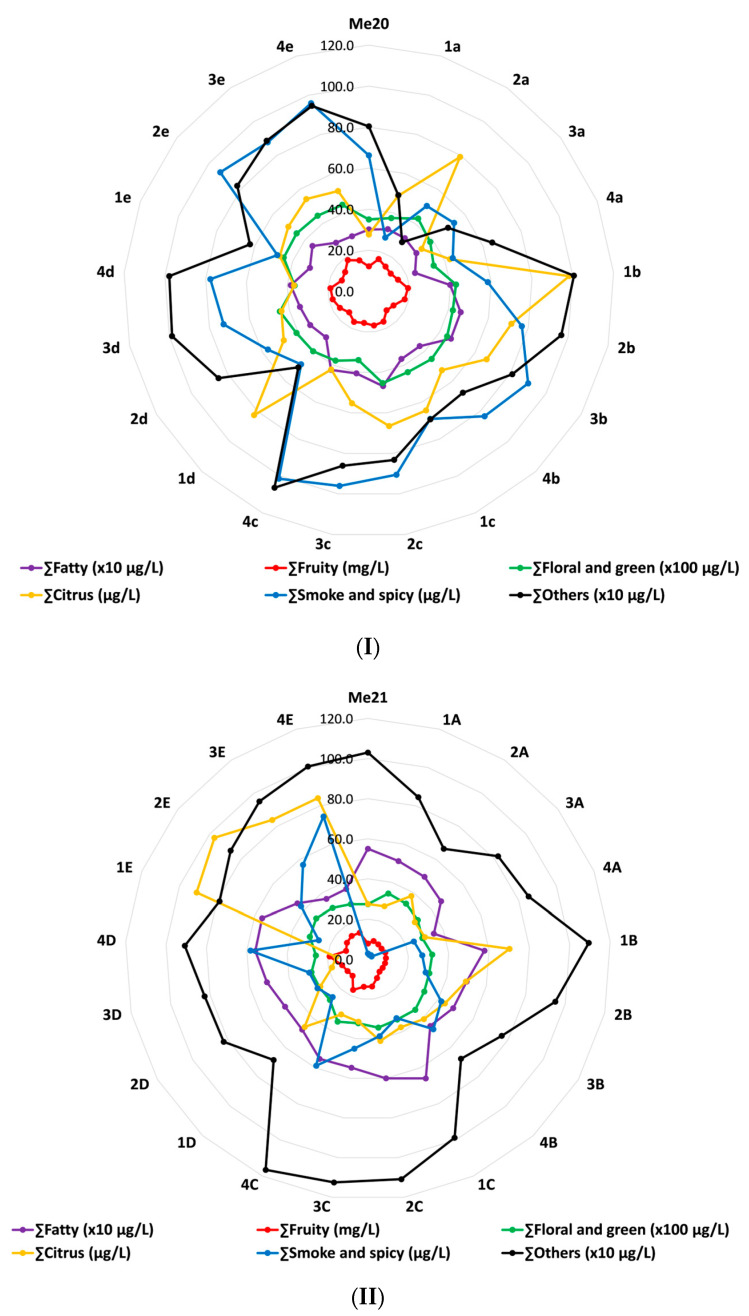
Total concentrations of main odour groups (fatty, fruity, floral and green, citrus, smoke and spicy and others) in vintage 2020 (**I**) and vintage 2021 (**II**) Merlot red wines and samples during their 12-month storage in different vessels. Abbreviations: (**I**) Me20—2020 vintage Merlot sample prior storage; a—stainless-steel tank; b—wooden barrel with excellent medium toasting; c—wooden barrel with excellent medium plus toasting; d—wooden barrel with excellent medium long toasting; e—wooden barrel with premium medium toasting; 1—sampling atin June 2021; 2—sampling atin September 2021; 3—sampling atin December 2021; 4—sampling atin March 2022. (**II**) Me21—2021 vintage Merlot sample prior storage; A—stainless-steel tank; B—wooden barrel with excellent medium toasting; C—wooden barrel with excellent medium plus toasting; D—wooden barrel with excellent medium long toasting; E—wooden barrel with premium medium toasting; 1—sampling in August 2022; 2—sampling in November 2022; 3—sampling in February 2023; 4—sampling in May 2023.

**Figure 3:**
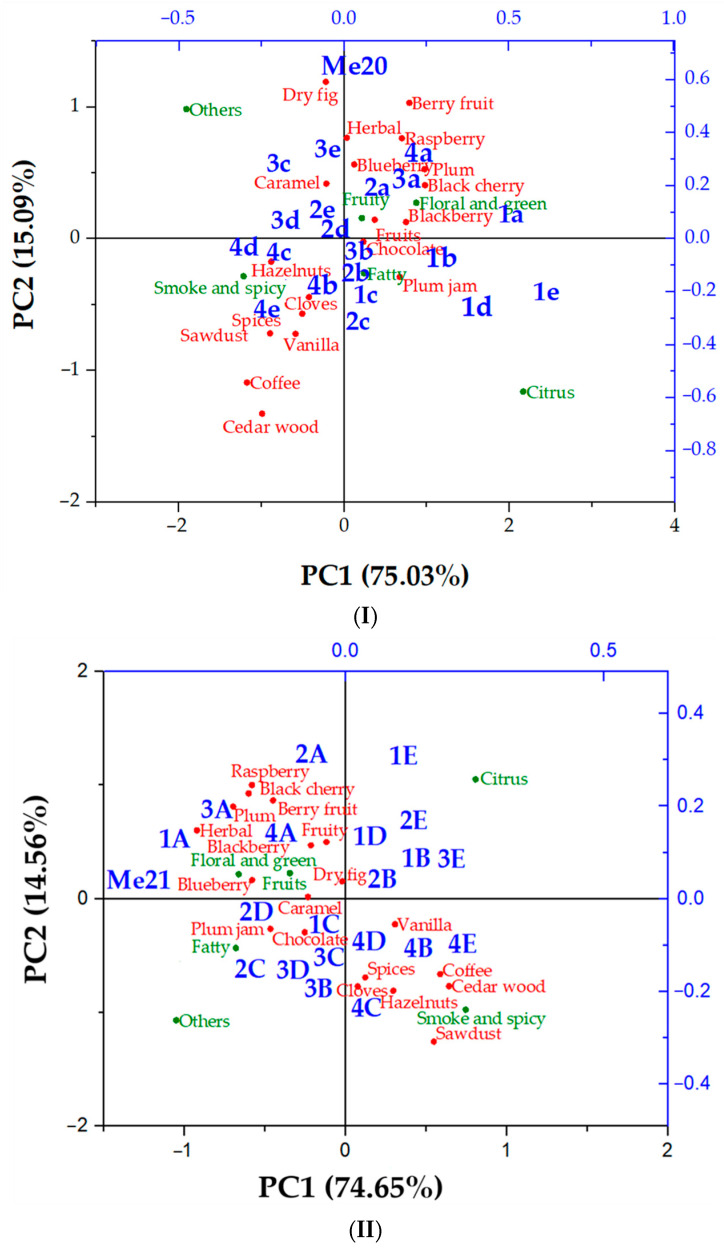
Principal component analysis (PCA) biplot of sensory descriptive analysis and GC/MS main aroma groups analysis in vintage 2020 (**I**) and vintage 2021 (**II**) Merlot red wine and samples obtained during 12-month storage in different vessels. Abbreviations: (**I**) Me20—2020 vintage Merlot sample prior storage; a—stainless-steel tank; b—wooden barrel with excellent medium toasting; c—wooden barrel with excellent medium plus toasting; d—wooden barrel with excellent medium long toasting; e—wooden barrel with premium medium toasting; 1—sampling atin June 2021; 2—sampling atin September 2021; 3—sampling atin December 2021; 4—sampling atin March 2022. (**II**) Me21—2021 vintage Merlot sample prior storage; A—stainless-steel tank; B—wooden barrel with excellent medium toasting; C—wooden barrel with excellent medium plus toasting; D—wooden barrel with excellent medium long toasting; E—wooden barrel with premium medium toasting; 1—sampling in August 2022; 2—sampling in November 2022; 3—sampling in February 2023; 4—sampling in May 2023. Red colour–samples; blue colour–groups of volatiles, green colour–aroma groups.

Merlot red wines (vintage 2020 and 2021) were produced under the same conditions, and the final product, after taking an initial sample, was stored in five different vessels: SST, EMT, EMT+, EMLT and PMT. The ageing process lasted for 12 months, and every 3 months, a sample from each vessel was taken for analysis. The analysis of samples involved gas chromatography with mass spectrometry (GC/MS) where the aroma profile was determined, and sensory evaluation and descriptive analysis of samples were performed with the help of a panel of trained evaluators. The results obtained are presented in Table 2, Table 3, Table 4, Table 5, Table 6, Table 7, Table 8, Table 9, Table 10 and Table 11 and Figure 1, Figure 2 and Figure 3.

Table 2 presents 49 aroma compounds identified by GC/MS in all mentioned samples, along with their retention time and index and main odour. Volatile compounds are divided into six groups: 6 volatile acids, 10 higher alcohols, 4 carbonyl compounds, 5 terpenes, 20 esters and 4 volatile phenols. They can also be divided into six groups according to their main odour: fatty, fruity, citrus, floral and green, smoke and spicy and others (vinegar, sulphurous, caramel and faint odour). The aroma profiles of 2020 (Me20) and 2021 (Me21) vintage Merlot were very similar. The differences in concentrations of individual aroma compounds were observed, although similar climate conditions throughout the year were recorded (Table 1). However, during August and September, the months of maturation and ripening of the grapes in the vineyard, in 2020, higher precipitation and similar sunshine hours were recorded compared to 2021.

Table 3 and Table 4 present the concentrations of volatile acids and phenols in 2020 and 2021 vintage Merlot samples obtained during their 12-month storage in different vessels. In analysed samples, six acids were identified (acetic, hexanoic, decanoic, lauric, myristic and palmitic acid). Hexanoic acid was not detected in any sample of 2020 vintage Merlot. The highest concentration among all acids was measured for acetic acid, with 631.0 μg/L in the initial Me20. The initial Me21 contained all mentioned acids, and the concentration of acetic acid was also the highest among acids (609.6 μg/L). After the first 3 months of ageing, the concentration of acetic acid decreased in all samples compared to the initial wine for both Me20 and Me21. However, during storage, an increase in the acetic acid concentration was observed in all samples, and the highest concentration was in Me20 and Me21 wine from EMT+, with 780.5 and 732.9 μg/L, respectively. The lowest concentrations were measured in both wines stored for 12 months in EMT: 438.7 μg/L in Me20 and 310.5 μg/L in Me21.

As mentioned, hexanoic acid was only detected in Me21 (31.5 μg/L), but its concentration decreased during the 12-month storage in each vessel, and the lowest concentrations were measured in wines stored in EMT and PMT, with 10.7 and 10.5 μg/L, respectively. SST was more favourable for preservation than wooden barrels, and the concentration of hexanoic acid after 12 months in SST was 19.2 μg/L.

The concentrations of the rest of the acids varied and differed between vintages and vessels. SST resulted in a decrease in decanoic acid after 12 months of storage of both wines, from 46.8 μg/L in the initial Me20 to 40.5 μg/L in SST, and from 241.5 μg/L in the initial Me21 to 113.3 μg/L in SST. Wooden barrels were more favourable for the retention of decanoic acid at the beginning of the storage, especially after 3 and 6 months. However, longer storage resulted in a decrease in the concentration of this acid. The highest concentrations of decanoic acid in final wines were measured in Me20 stored in the PMT barrel (65.4 μg/L) and in Me21 stored in EMT and EMLT barrels (148.4 and 146.6 μg/L, respectively).

In Me20 samples, the initial myristic acid concentration (2.7 μg/L) increased after storage, especially in EMT+ (9.5 μg/L). In Me21 samples, the initial concentration was 4.3 μg/L, but the highest concentration of myristic acid after 12 months of storage was measured in PMT (5.3 μg/L). A decrease in lauric acid was observed in the initial months of storage, but the concentrations of both wine vintages slightly increased during 12-month storage. Palmitic acid concentrations decreased in both wines stored in SST and increased after 3 months of storage in oak barrels with a downward trend during 12-month storage, except for the PMT barrel, where the highest concentrations were measured in both wines (19.8 μg/L in Me20 and 28.0 μg/L in Me21).

Table 3 and Table 4 also present the content of volatile phenols (4-ethylphenol, 4-ethylguaiacol, 4-propylguaiacol and 2,4-di-T-butylphenol) in analysed samples. In both initial wines, Me20 and Me21, 2,4-di-T-butylphenol was detected (46.8 and 37.0 μg/L, respectively) and its concentration increased after 12-month storage, regardless of the ageing vessel. However, the highest concentration of the mentioned compound in Me20 wine was found after 9-month storage in an EMT barrel (138.4 μg/L), but it then decreased, and after 12 months, the highest values were in EMT+ and PMT barrels (114.4 and 115.5 μg/L). On the other hand, the EMT barrel was more favourable for 2,4-di-T-butylphenol in Me21 than other vessels, and it resulted in the highest concentration after 12 months of storage (74.5 μg/L).

In Me21 wine, 4-propylguaiacol was not detected, regardless of the ageing vessel or storage time. This compound was also not detected in the initial Me20 wine and wine stored in SST and EMLT barrels, but small amounts were detected in Me20 wine stored in EMT, EMT+ and PMT barrels (5.7, 5.6 and 4.1 μg/L after 12 months of storage). 4-ethylguaiacol was not detected in the initial Me21 wine or after storage, except in samples obtained from the PMT barrel after 9 months (8.4 μg/L) and 12 months (12.3 μg/L) of storage. The same compound was initially present in Me20 (13.2 μg/L). Its concentration slightly decreased after the first month of storage in all vessels, especially in SST where the concentration was 3.8 μg/L, and it decreased over time and was, therefore, not detected in any other samples from SST. On the other hand, in EMT+, EMLT and PMT barrels, its concentration increased with storage time to 17.3, 14.0 and 15.7 μg/L, respectively. The concentration of 4-ethylphenol in the initial Me20 wine was 47.0 μg/L and its concentration decreased in SST, reaching 38.7 μg/L after 12 months of storage. In the initial Me21 wine and after its storage of 3, 6 and 9 months in SST, this compound was not detected. Only after 12-month storage in SST was 22.4 μg/L recorded. Further, 4-ethylphenol was found in all samples obtained from oak barrels, regardless of the vintage and storage time. The longer the storage time, the higher the concentration of 4-ethylphenol in all oak barrels and for both wines. The highest concentration of 4-ethylphenol for Me20 was measured in the EMT barrel (62.8 μg/L), and for Me21, in the PMT barrel (55.5 μg/L) after 12-month storage.

Table 5 and Table 6 present 10 higher alcohols (isoamyl alcohol, 2,3-butanediol, 1-hexanol, 1-heptanol, methionol, 2-ethyl-1-hexanol, benzyl alcohol, 1-octanol, 2-phenylethanol and dodecanol) and their concentrations measured in Me20 and Me21 and samples stored for 12 months in different vessels. Initial wines contained different concentrations of higher alcohols, and in Me20 samples, 1-heptanol was not detected, but it was detected in all Me21 samples. The initial concentration of 1-heptanol of 6.2 μg/L in Me21 changed during storage in different vessels, but after 12 months, its concentration was higher than the initial concentration in all vessels (10.8 μg/L in SST, 6.4 μg/L in EMT, 12.1 μg/L in EMT+ and 12.3 in EMLT barrel), except in the PMT barrel, where it was slightly decreased (5.7 μg/L).

The changes in alcohol concentrations during storage depended on the initial concentration of the corresponding alcohol, wine vintage and vessel type. The highest concentrations were measured for isoamyl alcohol (more than 10.0 mg/L in Me20 samples and more than 6.5 mg/L in Me21 samples) and 2-phenylethanol (more than 3.1 mg/L in Me20 samples and more than 2.5 mg/L in Me21 samples). The rest of the higher alcohols had concentrations around or lower than 1 mg/L in all samples.

In both wines, the initial concentration of isoamyl alcohol, 2-ethyl-1-hexanol, benzyl alcohol, 1-octanol, 2-phenylethanol, and dodecanol increased or did not significantly change after 12-month storage in different vessels. Except for 1-octanol whose concentration was the highest in SST, the oak barrels were more favourable for retention and increases in the concentration of other mentioned alcohols. However, the PMT barrel resulted in a slightly lower concentration of 1-octanol in Me21 after 12 months of storage compared to the initial concentration (19.6 μg/L to 13.1 μg/L). A similar trend was observed for dodecanol in the initial Me20 wine (58.1 μg/L) whose concentration decreased in SST and PMT barrels (35.0 and 55.7 μg/L, respectively).

The initial concentrations of 2,3-butanediol in Me20 and Me21 were different (664.1 and 416.4 μg/L, respectively), and the highest concentration of 2,3-butanediol in Me20 and Me21 was measured after 12-month storage in the EMLT barrel (664.2 and 1162.6 μg/L, respectively). The highest concentration of methionol in the mentioned wines was measured after 12-month storage in the EMT+ barrel (45.0 and 42.5 μg/L, respectively). The EMLT barrel, however, resulted in the lowest concentrations of methionol in both wines after 12-month storage.

The initial concentrations of 1-hexanol in Me20 and Me21 were 253.6 and 125.0 μg/L, respectively, and they varied during storage. The lowest concentrations of this compound after 12 months of storage in Me20 samples were found in the EMT+ barrel. A similar trend was observed for 1-hexanol in Me21 from the EMLT barrel. On the other hand, after 12 months of storage, the highest final concentration of 1-hexanol in Me21 was observed in the EMT+ barrel (256.5 μg/L). Among Me20 samples, the PMT barrel resulted in the highest concentration of 1-hexanol at the end of storage time (356.1 μg/L).

The content of carbonyl compounds (4-propylbenzaldehyde, geranyl acetone, lily aldehyde and hexyl cinnamaldehyde) and terpenes (linalool, hotrienol, β-citronellol, eugenol and β-damascenone) in Me20 and Me21 wines and samples stored for 12 months in SST and oak barrels are presented in Table 7 and Table 8. In the initial wines, the concentrations of carbonyl compounds differed between Me20 and Me21, and lily aldehyde was only not detected in the initial Me20 wine. The highest concentrations among all carbonyl compounds were measured for 4-propylbenzaldehyde in both initial wines (26.8 μg/L in Me20 and 12.5 μg/L in Me21). The concentration of all carbonyl compounds changed during 12-month storage, and mostly decreased in each vessel, except for the EMT+ barrel, where the highest final concentrations were measured in both wines. The PMT barrel resulted in the total loss of geranyl acetone in Me20 after only 3 months of storage, and this compound was not detected any further in this barrel.

Different storage vessels did not influence terpenes the same way in the two wine vintages. Eugenol was not detected in any initial wine or during storage in SST, but it was found in samples obtained from oak barrels with different toasting methods. The longer the storage time, the higher the concentration of eugenol, especially in Me20 from PMT barrel (11.1 μg/L), or in Me21 from EMT and EMT+ barrels (around 4.5 μg/L). The highest concentration of hotrienol was measured in EMT+ barrels of Me20 and Me21 wine (73.9 and 17.7 μg/L, respectively), where its concentration increased during storage. In the rest of the vessels, its concentration slightly increased at the beginning of the storage, but later, especially after 9 months, it started to decrease.

The concentration of linalool slightly increased during the first 3 months of storage, but later it started to decrease. However, the highest concentration was measured in Me21 in the EMT+ barrel (18.5 μg/L) and in Me20 in EMT 38.0 μg/L. In Me20 wine, the highest concentrations of β-citronellol and β-damascenone after 12 months of storage were measured in samples obtained from PMT barrels (7.3 and 8.0 μg/L, respectively). Other vessels resulted in a decrease in their concentrations during storage, and the lowest ones were measured in the EMLT barrel for β-citronellol (4.8 μg/L) and SST for β-damascenone (4.0 μg/L). On the other hand, in Me21 samples, a decrease in those compounds was observed during storage in each vessel. Nevertheless, SST and PMT barrels were more favourable for their retention than other barrels (7.3 μg/L was retained in both, that is, 68.9% of the initial concentration). Although an initial increment of β-damascenone in Me21 wine was observed after 3 months of storage, a downward trend was noticed in every vessel during longer storage time, with the highest final concentration measured in EMT+ barrel (9.3 μg/L), compared to the rest of samples.

Esters (Table 9 and Table 10) were the largest group among all six aroma groups identified in Merlot samples from two different vintages and five ageing vessels. Twenty esters were identified, and only ethyl cinnamate was not detected in any Me21 sample, unlike Me20 samples, where it was only not detected in samples from PMT barrels. Its initial concentration in Me20 wine (5.5 μg/L) did not significantly change or slightly increased at the beginning of the storage, but with storage time, its concentration decreased in all vessels. However, the highest final concentration after 12 months of storage was measured in the EMT+ barrel (5.8 μg/L).

Storage in the PMT barrel after 12 months also resulted in the lowest concentrations of many esters—ethyl hexanoate, phenethyl acetate, methyl palmitate, ethyl palmitate and ethyl stearate in samples from both vintages, and ethyl octanoate, ethyl hydrogen succinate, ethyl decanoate, ethyl vanillate, ethyl laurate, methyl dihydrojasmonate, diisobutyl phthalate and ethyl linoleate in Me21 samples. The SST also resulted in the lowest concentrations of most esters in both wines, compared to other vessels, especially ethyl myristate, isopropyl myristate, dibutyl phthalate and ethyl linoleate. In Me20, ethyl hydrogen succinate and methyl palmitate were not detected in the initial wine and samples from SST. On the other hand, SST was most favourable for ethyl octanoate retention in Me20 and Me21 wines stored in this vessel (210.7 and 233.5 μg/L, respectively) and ethyl stearate retention (39.1 and 17.7 μg/L, respectively) after 12-month storage.

The results showed that the Excellence barrels were most favourable for esters formation or retention in wine, and their concentrations depended mostly on the wine vintage, storage time and toasting method. Similar trends for some esters were observed for both wine vintages. For example, the EMT barrel showed the best results regarding ethyl 4-hydroxybutanoate, diethyl succinate and phenethyl acetate, where their concentrations were higher or not significantly different from the initial one. The highest concentrations among esters in samples from the EMT+ barrel were measured for ethyl hexanoate, methyl dihydrojasmonate, methyl palmitate and ethyl palmitate, for both wine vintages. The highest concentrations of ethyl hydrogen succinate and ethyl vanillate in Me20 and Me21 samples after 12 months of storage were found in the EMLT barrel. The initial concentration of ethyl vanillate increased after mentioned storage time in both samples. On the other hand, the concentration of ethyl hydrogen succinate, which was not detected in the initial Me20, increased to 157.8 μg/L in the EMLT barrel, but in the Me21 sample from the same barrel, the initial concentration of 238.5 μg/L did not significantly change.

In Table 11, the most abundant and marked compounds from each aroma group are separated, along with their initial concentrations in both wines and the highest concentrations obtained among the five vessels after 12 months of storage. Their descriptions and significance for the aroma of red wine are also presented in the table.

For easier comparison, the total concentrations of a group of the same type of compounds (acids, alcohols, carbonyl compounds, terpenes, esters and volatile phenols) were summarised and principal component analysis (PCA) was performed, as presented in Figure 1. In the first part of Figure 1(I), the PCA biplot for the 2020 vintage Merlot before and during storage in different vessels is shown. This PCA biplot was divided into PC1 with 56.87% and PC2 with 21.87% of the total variance. PC1 separated the biplot on samples obtained after 3 and 6 months of storage (negative side) and those obtained after 9 and 12 months of storage (positive side), and PC2 separated the biplot according to the content of terpenes, carbonyl compounds and acids (positive side) and alcohols, esters and volatile phenols (negative side). It can be observed that the initial Me20 wine (positive side of PC1 and PC2) is separated from the rest of the samples, meaning that changes in the aroma profile occurred during storage. The samples obtained after 3 and 6 months of storage in SST were also separated (negative side of PC1 and positive side of PC2), where the lowest concentrations of aroma compounds were mostly found. In the fourth quadrant, the positive side of PC1 and negative side of PC2 showing samples obtained after 9 and 12 months of storage in the PMT barrel were slightly separated from other samples, meaning that the most changes occurred in this barrel.

The second part of Figure 1(II) shows the PCA biplot of the 2021 vintage Merlot before and during storage in different vessels. PC1 (72.93% of total variance) separated the samples according to the dominant aroma compounds: alcohols, volatile phenols and esters on the negative side and acids, carbonyl compounds and terpenes on the positive side. PC2 (13.09%) separated the samples based on those obtained after 3- and 6-month storage (negative side) and those obtained after 9- and 12-month storage (positive side). Similar to Me20, Me21 was separated from other samples (positive side of PC1 and PC2). Also, samples obtained from PMT barrels after 9 and 12 months of storage were slightly separated from others. The rest of the samples were clustered in the middle of the PCA biplot with slight differences regarding storage vessel and time.

However, differences were observed between the two vintages of Merlot wine. In Me20 samples, it can be observed that alcohols and esters prevailed mostly during the initial months of storage in EMT+ and EMLT barrels, while terpenes prevailed in EMT, EMT+ and EMLT barrels, and slightly in SST during those months. Longer storage resulted in more carbonyl compounds and acids, especially in samples obtained from EMT and EMT+ barrels. In Me21, at the beginning of storage, terpenes and carbonyl compounds (especially after 3 months of storage) and esters (especially after 6 months of storage) prevailed in all samples. Longer storage of Me21 wine in SST and EMT+ barrels increased the total concentration of acids, but in EMT and EMLT, the total concentrations of alcohols and volatile phenols increased with storage time. Total volatile phenol concentrations also increased with storage time for 2020 vintage Merlot in all vessels, but their concentration change differed from Me21 samples.

As mentioned before and shown in Table 2, all aroma compounds identified in analysed samples were divided into six groups according to their main odour: fatty, fruity, citrus, floral and green, smoke and spicy and others (vinegar, sulphurous, caramel and faint odour). By summing up the concentrations of all compounds in each group, Figure 2 was obtained. It can be observed that the final concentration of each group of compounds depended on the initial concentration of each compound, wine vintage, storage time and vessel type.

In both wines, the concentration of fruity, citrus, floral and green aroma compounds increased during the initial months of storage in all vessels, but their concentration decreased with longer storage time. The exception was the fruity group in both wines, whose concentration increased with storage in the EMLT barrel. The content of compounds with a fatty odour in the 2021 vintage Merlot decreased during storage in all vessels, except in the EMLT barrel where an increasing trend was observed, but the final content was similar to the initial one. In the 2020 vintage Merlot, the fatty odour also showed a downward trend during storage in all vessels except in the EMLT barrel. However, the final concentrations of the mentioned group of compounds in samples from Excellence barrels were higher than the initial one.

The aroma group named Others contained acetic acid, methionol, ethyl 4-hydroxybutanoate, ethyl hydrogen succinate, 4-propylbenzaldehyde, isopropyl myristate, diisobutyl phthalate, dibutyl phthalate and 2,4-di-T-butyl phenol. Except in samples obtained from EMT barrels, a growing trend of their concentrations was observed in other vessels during the 12-month storage of both wines. The EMT+ barrel was most favourable for this group of compounds, regardless of the wine vintage.

The concentrations of compounds with smoke and spicy odours increased during the storage of both wines in all wooden barrels, especially Me20 in the EMT+ barrel and Me21 in the PMT barrel, where the highest final concentrations were measured. The stainless-steel tank was not favourable for this aroma group.

Trained sensory evaluators performed the sensory evaluation of all samples and their results are presented in Table 12 and Table 13. According to the 100-point test, all samples received more than 80 points, except the Me20 obtained from the EMLT barrel after 12 months of storage, which received the lowest points of 79.7. In summary, the samples from SST received lower points than the ones from wooden barrels. The highest points among Me20 samples were awarded to the sample from the EMT barrel (95.0), but only after 3 months of storage. Longer storage in this barrel resulted in lower points. However, if 12-month storage is taken into account, Me20 from the PMT barrel received the highest points (91.3). Further, the Me21 sample from the PMT barrel after 12 months of storage received the highest points among all samples (95.7).

The sensory evaluators also conducted a descriptive analysis, where the following aromatic notes were evaluated: plum, black cherry, raspberry, berry fruits, blackberry, blueberry, plum jam, dry fig, chocolate, cedar wood, sawdust, cloves, vanilla, caramel, coffee, hazelnuts, spices, fruits and herbal. Each note was rated with points from 0 to 10, where 10 was the highest intensity. These results were compared to the content of aroma groups obtained from gas chromatography with mass spectrometry (GC/MS), and Figure 3 was obtained. In both parts of Figure 3, PC1 accounts for around 75% and PC2 for around 15% of total variance. In the first part of Figure 3, samples of 2020 vintage Merlot are presented. On the positive side of PC1, samples obtained in the third and sixth months were placed, and on the negative side of PC1, the ones obtained in the ninth and twelfth months of storage (except SST) were placed. PC2 separated them based on the samples with dominating fruity, floral and green aromas (positive side) and the ones with dominating smoke, spicy, fatty and citrus aromas (negative side). However, each quadrant contained one group of samples that were clustered together along with certain aromatic notes. Fruity, floral and green were clustered together with samples obtained from SST. For those samples, sensory evaluators gave the most points for blackberry, black cherry, blueberry, raspberry, plum and herbal notes. Smoke and spicy aromas prevailed in samples obtained from all wooden barrels after 12 months of storage, and sensory evaluators found mostly hazelnut, clove, spice, sawdust and vanilla notes. Shorter storage (mostly 3 or 6 months) in wooden barrels resulted in plum jam and chocolate notes, which were clustered with compounds contributing to the fatty aroma. The aroma groups citrus (quadrant I) and others (quadrant II) were separate from the rest of the samples. All samples were separate from the initial Me20, meaning that the aroma profile changed significantly, which is consistent with the results obtained by GC/MS and presented in Table 3, Table 4, Table 5, Table 6, Table 7, Table 8, Table 9, Table 10 and Table 11.

Similar results can be observed in the second part of Figure 3, where samples of the 2021 vintage Merlot are presented. The citrus and others groups were separated from the rest of the aroma groups. Although the initial sample of Me21 was separate from other samples, the sample stored for 3 months in SST had a very similar aroma to the initial wine. Further, SST resulted in intensive floral and green aromas, accompanied by plum, berry fruits and herbal notes described by sensory evaluators. Samples from EMT, EMT+ and EMLT after 6 or 9 months of storage were clustered with caramel, plum jam, chocolate and fatty aromas. Longer storage (12 months) in all wooden barrels resulted in smoke and spicy aromas that sensory evaluators described as vanilla, coffee, cloves, hazelnuts, sawdust and cedar wood.

**Figure 4:**
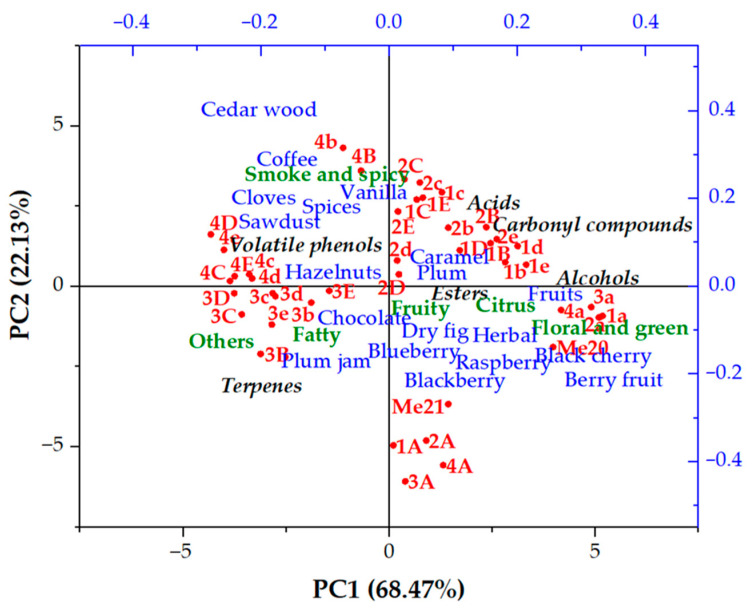
Principal component analysis (PCA) biplot of sensory descriptive analysis and GC/MS main aroma groups analysis in vintage 2020 and vintage 2021 Merlot red wine and samples obtained during 12-month storage in different vessels. Abbreviations: Me20—2020 vintage Merlot sample prior storage; a—stainless-steel tank; b—wooden barrel with excellent medium toasting; c—wooden barrel with excellent medium plus toasting; d—wooden barrel with excellent medium long toasting; e—wooden barrel with premium medium toasting; 1—sampling atin June 2021; 2—sampling atin September 2021; 3—sampling atin December 2021; 4—sampling atin March 2022; Me21—2021 vintage Merlot sample prior storage; A—stainless-steel tank; B—wooden barrel with excellent medium toasting; C—wooden barrel with excellent medium plus toasting; D—wooden barrel with excellent medium long toasting; E—wooden barrel with premium medium toasting; 1—sampling in August 2022; 2—sampling in November 2022; 3—sampling in February 2023; 4—sampling in May 2023. Red colour–samples; black colour–groups of volatiles, green colour–aroma groups, blue colour–aromatic notes.

For a comparison of chemical and sensory properties between two vintages, a PCA biplot was created according to the results obtained by GC/MS and sensory evaluators (Figure 4). PC1 (68.47% of total variance) separated the samples based on those obtained after 3 and 6 months of storage (positive side) and those obtained after 9 and 12 months of storage (negative side). PC2 (22.13% of total variance) separated the samples based on those with more pronounced volatile phenols, smoke and spicy aromas, then acids, carbonyl compounds and alcohols (positive side) and those with more pronounced terpenes and esters, along with fruity, citrus and floral aromas. However, all quadrants could be observed separately. In the first quadrant (negative side of PC1 and positive side of PC2), samples obtained after 12 months of storage were clustered, and volatile phenols, spices and smoke aromas prevailed. This was observed for both Merlot vintages. Samples obtained after 9 months of storage of both wine vintages had more fatty aromas with chocolate and plum jam notes. Acids, carbonyl compounds and alcohols prevailed in the initial months (3 and 6 months) of storage in wooden barrels. On the other hand, samples from stainless-steel tanks were separated in the fourth quadrant (positive side of PC1 and negative side of PC2), with more pronounced fruity and floral aromas of esters. Those samples were also separated according to the vintage, and they were clustered near the corresponding initial Merlot wine, meaning that production conditions during different years had a great influence on the Merlot aroma. However, this PCA biplot showed that similar changes in aroma profiles occurred in both vintages of Merlot wine during the 12 months of storage in different vessels.

## 4. Discussion

Ageing and storage are important stages of the wine vinification process. Different factors influence this stage: time, temperature, vessel type, wine type, oxygen presence, etc. Red wine is usually stored in wooden barrels that react with wine, yielding different compounds and resulting in a unique and complex aroma [8]. Culleré et al. [30] investigated the most odour-active compounds in acacia, chestnut, cherry, ash and oak woods. Their results showed that each type of wood had its own characteristic aroma profile, and many of the identified compounds transferred into wine or reacted with compounds in wine during storage. Besides the type of wood, an important factor during wine ageing is the barrel size and the surface of wood and wine contact. A previous study of different sizes of wood pieces (chips and staves) with different toasting levels (light, medium and heavy) indicated that both have a significant influence: the size influenced the extraction kinetics and the toasting level resulted in different types and concentrations of aroma compounds in wine [31]. The use of wood chips or old barrel fragments with different toasting levels has also been investigated as an alternative to ageing in wooden barrels. It has been shown that wooden chips could be used for short-aged wines and to reuse wood from old barrels, but new wooden barrels result in more complex aromas and higher wine quality [32], although several other factors must also be taken into account, such as the toasting level, wine type, wood type, barrel age, contact surface between wood and wine and other [25].

In this study, the influence of different ageing vessels on Merlot red wine during 12 months of storage was investigated. For that purpose, Merlot wine was produced from 2020 and 2021 vintage Merlot grapes, and five different vessels for ageing and storage were used: SST, EMT, EMT+, EMLT and PMT. Samples were taken every 3 months during 1 year of storage. For aroma profile analysis, gas chromatography with mass spectrometry was used (GC/MS) and a sensory evaluation was conducted.

The results showed that different storage vessels influenced the final aroma of Merlot wine. Aroma profiles of 2020 and 2021 vintage Merlot were very similar, and the same aroma compounds were identified with GC/MS. However, there were differences in their concentrations due to different climate conditions during the mentioned years and harvest dates, as expected [33]. Different initial concentrations of aroma compounds and their mutual interactions and interactions with the vessel surface have resulted in different final aromas of both wine vintages, comparing the same vessels [34]. However, in summary, there were also some great similarities in aroma changes in two wines stored in the same vessel. González-Centeno et al. [19] investigated the influence of the toasting method on three different wines. They concluded that toasting level has a great impact on wine aroma and chemical composition, resulting in an increase in similar combinations of compounds but different extraction rates in different wines. In this study, for example, in both analysed wine vintages, smoke and spicy notes increased in wooden barrels, unlike in SST, where floral and fruity aromas prevailed because of the lack of smoke and spicy aromas.

All identified aroma compounds in this study were divided into six groups: acids, alcohols, carbonyl compounds, terpenes, esters and aroma phenols. Volatile acids in wine represent a group of mostly fatty acids that contribute to the wine’s aroma. Acetic acid is a representative of this group and it contributes to the wine aroma if its concentration does not exceed 0.9 g/L; otherwise, it leads to a vinegar-like taste and wine spoilage [35]. It is formed partially by yeasts during fermentation in anaerobic conditions, and later by acetic acid bacteria in aerobic conditions as a result of ethanol oxidation. Oxygen is naturally present in wine, but during wine storage in wooden barrels, oxygen permeates through barrel staves. The amount of oxygen that permeates in wine depends on the type of barrels, wood type, grain density of staves, etc. [36]. This could explain the increase in acetic acid concentration during storage.

Besides acetic acid bacteria, small amounts of oxygen support the survival of *Brettanomyces* yeasts, especially in wooden barrels [22,37]. These yeasts produce 4-ethylphenol, 4-ethylguaiacol and similar compounds that, in excess concentrations, contribute to the undesirable aroma with medicinal, horse sweat or stable notes. On the other hand, if their concentrations are below the undesirable threshold (several hundred μg/L for 4-ethylphenol and 50 to 100 μg/L for 4-ethylguaiacol), they could contribute to a pleasant mild smoky aroma [1,38].

Although these compounds were not detected in the initial 2021 vintage Merlot in this study, 4-ethylphenol was detected in all samples from wooden barrels and 4-ethylguaicol in samples from the Premium barrel with medium toasting after 9 and 12 months of storage. The initial 2020 vintage Merlot contained both compounds, and their concentrations slightly increased during storage in wooden barrels. Nevertheless, in all samples, those concentrations were lower than the mentioned threshold, regardless of their concentration increment during storage. The SST was not favourable for their retention after 12 months of storage and resulted in their loss. Furthermore, the results from a previous study [39] showed that storage time had a higher influence on volatile phenols than oenological parameters or barrel type.

Higher alcohols are usually the most abundant group of aroma compounds, and they contribute to the wine aroma if their concentrations are lower than 400 mg/L [40]. In this study, storage time and vessel type influenced differently on individual higher alcohols in both wine vintages, but in summary, an increase in total alcohol concentration was observed compared to the initial concentration. Higher alcohols are formed by yeast’s amino acid metabolism or by the reduction in related aldehydes during fermentation. They could also be formed during the ageing and storage of wine, mostly as a result of ester hydrolysis or evaporation during wood maturation [16].

Esters in wine represent an important, highly aromatic and the most numerous group of volatiles. They are divided into esters formed during fermentation and the ones formed during wine ageing and storage [41]. During fermentation, they are formed via the esterification of fatty acids and a corresponding alcohol. Ethyl esters prevail because ethanol is the most abundant alcohol in wine [42,43]. In this study, storage time, vessel type and wine vintage had different influences on the concentration of esters. The decrease in their concentration usually occurs due to their hydrolysis or oxidation under the influence of oxygen and temperature [44,45]. However, some ester concentrations have increased during storage. Previous studies showed that certain polyphenols, like caffeic and gallic acid in wine, can inhibit the loss of esters or increase their stability through hydrogen bonding [46,47,48].

Carbonyl compounds (aldehydes and ketones) are usually present in small concentrations in wine, but they have a great influence on wine aroma. They are produced mostly during alcoholic fermentation and they usually have a sharp fruity or floral aroma [42]. The increase in carbonyl compound concentration could occur due to their extraction from wooden barrels or alcohol oxidation into aldehydes during storage. However, they could also react with other compounds in wine like polyphenols (they initiate the formation of condensed tannins and stabilization of colour compounds) and acids (formation of esters), resulting in a decrease in their concentration [49,50].

One of the mentioned aroma groups found in wines is terpenes. Each variety of grapes and wine has a unique combination of terpenes. They are highly aromatic, contributing to fruity and floral aromas, and are usually present in wine in concentrations below 1 mg/L [3]. During the storage of Merlot wines in this study, the concentrations of terpenes changed differently, depending mostly on vessel type and storage time. The increase in terpene concentrations during the storage of wine in wooden barrels usually occurs due to the extraction of these compounds from wood (especially eugenol, which contributes to the spicy aroma) [9,49]. However, the oxidation of terpenes and the phenomena of the sorption of aroma compounds by barrel staves have also been investigated, but further research is still necessary [51,52].

The sensory evaluation of wine is one of the most important analyses of wine because it is the most accepted and easy to understand for consumers. It is divided into three main parts: visual perception, olfactory and mouth-feel sensation [53]. There are several tests used for sensory evaluation, and they are performed by human wine experts. The most widely applied test is the 100-point test (OIV), and it was used in this study along with a descriptive analysis of samples. According to the results obtained from sensory evaluators, longer storage (more than 6 months) of Merlot in wooden barrels was not always recommended regarding wine aroma, because significant changes occurred. However, both wine vintages obtained from the Premium oak barrel with medium toasting after 12 months of storage received the highest points from evaluators. The main difference between Excellence and Premium barrels was the grain density. The results showed that this also affects the extraction of aroma compounds from wood to wine, microoxygenation, and the formation of new compounds [11]. Although all samples received high points (above 80.0), generally, the lowest points, regardless of storage time, were assigned to the samples from the stainless-steel tank. The descriptive analysis confirmed the results of gas chromatography: the lack of woody and spicy aromas in samples from stainless steel, which was not favourable for Merlot red wine, and the increase in these aromas in samples stored in wooden barrels with different toasting methods.

## 5. Conclusions

The main subject of this study was Merlot red wine ageing in a stainless-steel tank and four different barrels, and differences between two vintages of the same wine. According to the results of this study, various factors influenced the final wine aroma profile. The change in aroma compound content depended on the initial wine composition (differences between vintages were observed), vessel type (differences between samples from the stainless-steel tank and wooden barrels were observed), toasting method, grain density and storage time. Similarities and constant trends in changes in aroma compound concentrations were harder to notice if individual compounds were observed. According to the PCA analysis of the total concentrations of acids, alcohols, carbonyl compounds, terpenes, esters and volatile phenols, significant changes in the aroma profiles of both Merlot vintages occurred during storage in all vessels. The PCA analysis also showed that storage of Merlot in wooden barrels resulted in an increase in aroma compounds with smoke and spicy notes. This is consistent with the descriptive analysis of sensory evaluators, which described the aroma profile of these wines with spicy, cloves, coffee, cedar wood and vanilla notes. According to the 100-point test, the best-rated 2020 and 2021 vintage Merlot wines after 12 months of storage were obtained from Premium oak barrels with medium toasting.

This study showed that various factors should be included if the aim is to obtain a certain aroma profile of red wine. Even slight differences in the time and temperature of barrel toasting played a great role in the final Merlot aroma. Although this study provided useful information about the ageing process of red wine, further investigations including other types of vessels or wine varieties could be conducted.

## Figures and Tables

**Table 1:** Average monthly values of air temperature, sunshine hours and precipitation in vineyard Kutjevo during years 2020 and 2021 (Croatian Meteorological and Hydrological Service).

Month	Air Temperature (°C)		Sunshine Hours (h)		Precipitation (mm)	
	2020	2021	2020	2021	2020	2021
January	1.8	3.0	128.4	87.2	36.7	60.8
February	7.6	6.5	144.6	138.8	48.8	37.4
March	8.2	6.9	171.0	168.3	61.8	50.2
April	13.1	9.4	299.2	176.3	12.6	70.9
May	15.2	14.6	215.8	230.3	82.5	85.1
June	19.8	22.5	254.1	360.0	59.1	36.1
July	22.0	24.3	332.1	313.5	76.9	93.8
August	22.5	22.0	289.3	291.1	103.2	45.9
September	18.9	18.1	235.6	237.9	57.6	23.1
October	13.2	10.8	150.2	165.6	115.4	57.5
November	6.3	6.8	55.8	64.8	34.1	83.6
December	3.9	3.5	22.2	75.3	68.1	94.3
Total (year)	12.7	12.4	2298.3	2309.0	756.8	738.7

**Table 2:** Retention time, retention index and main odour of aroma compounds identified in 2020 and 2021 vintage Merlot and samples obtained during 12-month storage in different vessels.

Compound	RT *	RI **	CAS Number	Odour	Compound	RT *	RI **	CAS Number	Odour
Acids					Esters				
Acetic acid	3.62	722	64-19-7	vinegar	Ethyl hexanoate	18.69	997	123-66-0	fruity
Hexanoic acid	19.52	1011	142-62-1	fatty	Ethyl 4-hydroxybutanoate	22.35	1060	999-10-0	caramel
Decanoic acid	38.13	1380	334-48-5	fatty	Diethyl succinate	29.14	1188	123-25-1	fruity
Lauric acid	42.15	1562	143-07-7	fatty	Ethyl octanoate	29.83	1201	106-32-1	fruity
Myristic acid	44.81	1756	544-63-8	fatty	Ethyl hydrogen succinate	30.26	1157	1070-34-4	faint
Palmitic acid	47.44	1997	57-10-3	fatty	Phenethyl acetate	32.51	1251	103-45-7	floral
Alcohols					Ethyl decanoate	38.55	1391	110-38-3	fruity
Isoamyl alcohol	4.01	743	123-51-3	fruity	Ethyl cinnamate	40.19	1458	103-36-6	fruity
2,3-butanediol	6.19	819	513-85-9	fruity	Ethyl vanillate	42.45	1581	617-05-0	smoke
1-hexanol	9.45	869	111-27-3	green	Ethyl laurate	42.55	1587	106-33-2	fatty
1-heptanol	16.79	974	111-70-6	green	Methyl dihydrojasmonate	43.47	1651	24,851-98-7	floral
Methionol	17.26	980	505-10-2	sulphurous	Ethyl myristate	45.17	1784	124-06-1	fatty
2-ethyl-1-hexanol	20.64	1031	104-76-7	citrus	Isopropyl myristate	45.50	1815	110-27-0	faint
Benzyl alcohol	20.72	1032	100-51-6	fruity	Diisobutyl phthalate	46.07	1866	84-69-5	faint
1-octanol	23.13	1072	111-87-5	green	Methyl palmitate	46.58	1914	112-39-0	fatty
2-phenylethanol	25.39	1106	60-12-8	floral	Dibutyl phthalate	47.07	1961	84-74-2	faint
Dodecanol	40.39	1467	112-53-8	fatty	Ethyl palmitate	47.32	1984	628-97-7	fatty
Carbonyl compounds					Ethyl linoleate	48.98	2166	544-35-4	fatty
4-propylbenzaldehyde	33.15	1266	28,785-06-0	faint	Ethyl oleate	49.05	2167	111-62-6	fatty
Geranyl acetone	39.92	1446	3796-70-1	floral	Ethyl stearate	49.21	2185	111-61-5	fatty
Lily aldehyde	41.42	1517	80-54-6	floral	Volatile phenols				
Hexyl cinnamaldehyde	44.69	1746	101-86-0	floral	4-ethyl phenol	28.46	1174	99,123-07-9	smoke
Terpenes					4-ethyl guaiacol	33.49	1272	2785-89-9	smoke
Linalool	24.59	1094	78-70-6	citrus	4-propyl guaiacol	37.42	1363	2785-87-7	spicy
Hotrienol	24.92	1099	20,053-88-7	floral	2,4-di-T-butyl phenol	41.24	1506	96-76-4	faint
β-citronellol	31.39	1232	106-22-9	citrus					
Eugenol	37.15	1355	97-53-0	spicy clove					
β-damascenone	38.31	1385	23,726-93-4	fruity					

* retention time (min). ** retention index.

**Table 3:** Concentrations of acids and volatile phenols (µg/L) in aroma profile of 2020 vintage Merlot and samples obtained during 12-month storage in different vessels. Different superscript letters (a–o) in the same column indicate statistical differences determined by ANOVA and Fisher’s (LSD) test with *p* < 0.05.

Sample	Acids						Volatile Phenols			
	Acetic Acid	Hexanoic Acid	Decanoic Acid	Lauric Acid	Myristic Acid	Palmitic Acid	4-ethylphenol	4-ethylguaiacol	4-propylguaiacol	2,4-di-T-butylphenol
Me20	631.0 ± 12.3 ^l^	-	46.8 ± 0.8 ^bc^	9.4 ± 0.2 ^g^	2.7 ± 0.1 ^a^	11.6 ± 0.1 ^e^	47.0 ± 0.6 ^e^	13.2 ± 0.5 ^c^	-	46.8 ± 0.6 ^e^
1a	301.7 ± 8.1 ^c^	-	47.0 ± 0.5 ^c^	7.9 ± 0.3 ^de^	4.8 ± 0.2 ^c^	9.7 ± 0.1 ^d^	38.1 ± 0.7 ^b^	3.8 ± 0.1 ^a^	-	58.1 ± 1.0 ^g^
2a	160.7 ± 0.8 ^a^	-	47.2 ± 0.8 ^c^	8.2 ± 0.6 ^ef^	4.8 ± 0.1 ^c^	3.4 ± 0.3 ^a^	44.4 ± 0.5 ^d^	-	-	31.6 ± 0.8 ^a^
3a	298.5 ± 4.5 ^c^	-	47.4 ± 0.6 ^c^	8.0 ± 0.1 ^e^	4.8 ± 0.1 ^c^	3.8 ± 0.1 ^a^	47.9 ± 0.3 ^e^	-	-	96.7 ± 1.4 ^l^
4a	466.8 ± 0.9 ^f^	-	40.5 ± 0.1 ^a^	7.2 ± 0.1 ^d^	4.6 ± 0.1 ^c^	5.0 ± 0.5 ^b^	38.7 ± 0.4 ^b^	-	-	90.3 ± 1.2 ^j^
1b	386.0 ± 14.4 ^d^	-	102.9 ± 2.0 ^l^	8.7 ± 0.1 ^f^	6.2 ± 0.3 ^e^	23.1 ± 0.2 ^m^	42.3 ± 0.6 ^c^	-	3.4 ± 0.1 ^a^	51.9 ± 0.1 ^f^
2b	399.9 ± 2.1 ^d^	-	98.0 ± 0.2 ^k^	10.6 ± 0.4 ^h^	8.2 ± 0.1 ^g^	23.7 ± 0.7 ^m^	53.5 ± 0.3 ^g^	-	5.7 ± 0.1 ^d^	65.8 ± 1.6 ^h^
3b	384.9 ± 4.7 ^d^	-	76.2 ± 0.5 ^g^	11.2 ± 0.2 ^h^	6.3 ± 0.1 ^e^	10.0 ± 0.3 ^d^	68.3 ± 0.8 ^k^	-	5.5 ± 0.1 ^d^	138.4 ± 0.2 ^o^
4b	438.7 ± 6.6 ^e^	-	54.9 ± 0.2 ^d^	8.4 ± 0.2 ^ef^	7.5 ± 0.2 ^f^	6.9 ± 0.2 ^c^	62.8 ± 0.7 ^j^	-	5.7 ± 0.2 ^d^	95.1 ± 0.7 ^l^
1c	432.9 ± 5.7 ^e^	-	92.0 ± 0.3 ^j^	8.7 ± 0.1 ^f^	5.4 ± 0.4 ^d^	13.8 ± 0.2 ^g^	52.0 ± 0.8 ^g^	-	3.0 ± 0.1 ^a^	37.7 ± 0.1 ^c^
2c	504.9 ± 0.6 ^g^	-	108.1 ± 0.3 ^m^	8.4 ± 0.2 ^ef^	8.7 ± 0.1 ^g^	13.2 ± 0.1 ^g^	56.6 ± 1.5 ^h^	10.8 ± 0.1 ^b^	4.2 ± 0.2 ^bc^	45.6 ± 0.9 ^e^
3c	570.5 ± 6.2 ^j^	-	66.1 ± 0.5 ^f^	8.7 ± 0.2 ^f^	8.1 ± 0.1 ^g^	6.8 ± 0.2 ^c^	59.3 ± 0.4 ^i^	14.8 ± 0.1 ^d^	4.6 ± 0.1 ^c^	93.5 ± 0.2 ^k^
4c	780.5 ± 12.4 ^m^	-	62.4 ± 0.3 ^e^	8.4 ± 0.3 ^ef^	9.5 ± 0.1 ^h^	6.5 ± 0.2 ^c^	61.3 ± 0.7 ^j^	17.3 ± 0.1 ^f^	5.6 ± 0.1 ^d^	114.4 ± 1.3 ^n^
1d	229.8 ± 7.9 ^b^	-	88.4 ± 0.7 ^i^	7.5 ± 0.3 ^de^	5.3 ± 0.1 ^d^	20.2 ± 0.3 ^l^	34.6 ± 0.5 ^a^	4.2 ± 0.1 ^a^	-	39.6 ± 1.1 ^d^
2d	540.0 ± 0.2 ^i^	-	47.2 ± 0.9 ^c^	5.8 ± 0.3 ^b^	4.0 ± 0.1 ^b^	18.0 ± 0.2 ^j^	34.0 ± 0.1 ^a^	10.9 ± 0.5 ^b^	-	33.5 ± 0.1 ^b^
3d	618.5 ± 4.1 ^l^	-	45.9 ± 0.1 ^b^	7.7 ± 0.1 ^de^	6.2 ± 0.1 ^e^	12.6 ± 0.1 ^f^	43.8 ± 0.9 ^c^	14.4 ± 0.1 ^d^	-	77.1 ± 2.3 ^i^
4d	627.2 ± 5.7 ^l^	-	62.7 ± 0.2 ^e^	8.6 ± 0.1 ^f^	6.1 ± 0.1 ^e^	12.5 ± 0.1 ^f^	48.3 ± 0.8 ^f^	14.0 ± 0.4 ^d^	-	72.8 ± 2.3 ^i^
1e	439.5 ± 8.0 ^e^	-	92.8 ± 0.4 ^j^	5.1 ± 0.3 ^b^	4.8 ± 0.2 ^c^	17.0 ± 0.4 ^i^	43.3 ± 0.3 ^c^	10.3 ± 0.6 ^b^	-	32.2 ± 0.3 ^a^
2e	521.0 ± 1.3 ^h^	-	93.1 ± 2.0 ^j^	5.7 ± 0.5 ^b^	6.9 ± 0.4 ^ef^	15.5 ± 0.2 ^h^	47.8 ± 0.2 ^e^	15.9 ± 0.1 ^e^	3.9 ± 0.1 ^b^	47.1 ± 0.4 ^e^
3e	544.8 ± 4.2 ^i^	-	83.0 ± 0.2 ^h^	4.3 ± 0.1 ^a^	5.2 ± 0.1 ^d^	17.7 ± 0.1 ^ij^	48.8 ± 0.1 ^f^	15.8 ± 0.6 ^e^	3.9 ± 0.1 ^b^	109.2 ± 0.1 ^m^
4e	596.3 ± 1.4 ^k^	-	65.4 ± 0.6 ^f^	6.7 ± 0.2 ^c^	5.3 ± 0.1 ^d^	19.8 ± 0.1 ^k^	57.9 ± 0.1 ^h^	15.7 ± 0.1 ^e^	4.1 ± 0.1 ^b^	115.5 ± 0.1 ^n^

“-“ not detected. Abbreviations: Me20—2020 vintage Merlot sample prior storage; a—stainless-steel tank; b—wooden barrel with excellent medium toasting; c—wooden barrel with excellent medium plus toasting; d—wooden barrel with excellent medium long toasting; e—wooden barrel with premium medium toasting; 1a–1e—sampling in June 2021; 2a–2e—sampling in September 2021; 3a–3e—sampling in December 2021; 4a–4e—sampling in March 2022.

**Table 4:** Concentrations of acids and volatile phenols (µg/L) in aroma profile of 2021 vintage Merlot and samples obtained during 12-month storage in different vessels. Different superscript letters (a–n) in the same column indicate statistical differences determined by ANOVA and Fisher’s (LSD) test with *p* < 0.05.

Sample	Acids						Volatile Phenols			
	Acetic Acid	Hexanoic Acid	Decanoic Acid	Lauric Acid	Myristic Acid	Palmitic Acid	4-ethylphenol	4-ethylguaiacol	4-propylguaiacol	2,4-di-T-butylphenol
Me21	609.6 ± 10.2 ^k^	31.5 ± 0.1 ^k^	241.5 ± 3.2 ^k^	11.8 ± 0.2 ^h^	4.3 ± 0.1 ^c^	28.1 ± 0.5 ^l^	-	-	-	37.0 ± 0.7 ^c^
1A	419.7 ± 3.0 ^e^	33.8 ± 0.3 ^l^	199.1 ± 4.2 ^ij^	11.5 ± 0.1 ^h^	5.4 ± 0.1 ^e^	24.9 ± 0.7 ^j^	-	-	-	41.6 ± 0.4 ^e^
2A	303.1 ± 7.4 ^b^	14.9 ± 0.5 ^de^	200.0 ± 0.6 ^j^	13.6 ± 0.5 ^j^	5.5 ± 0.1 ^e^	16.9 ± 0.2 ^d^	-	-	-	32.9 ± 0.9 ^c^
3A	446.7 ± 6.0 ^f^	15.1 ± 0.2 ^e^	200.6 ± 0.6 ^j^	8.1 ± 0.4 ^e^	5.5 ± 0.3 ^e^	20.9 ± 0.5 ^g^	-	-	-	52.4 ± 0.8 ^h^
4A	505.4 ± 5.5 ^g^	19.2 ± 0.5 ^g^	113.3 ± 0.4 ^a^	6.0 ± 0.1 ^b^	4.5 ± 0.2 ^cd^	22.5 ± 0.3 ^h^	22.4 ± 0.6 ^d^	-	-	50.0 ± 0.3 ^g^
1B	267.8 ± 3.5 ^a^	29.3 ± 0.4 ^j^	252.9 ± 0.7 ^l^	8.7 ± 0.1 ^e^	4.9 ± 0.1 ^d^	29.9 ± 0.6 ^m^	20.0 ± 0.3 ^b^	-	-	40.6 ± 0.2 ^d^
2B	245.9 ± 4.2 ^a^	12.7 ± 0.2 ^b^	163.8 ± 0.6 ^f^	9.8 ± 0.1 ^f^	6.9 ± 0.1 ^f^	29.1 ± 0.8 ^m^	21.4 ± 0.1 ^c^	-	-	66.3 ± 0.2 ^k^
3B	320.6 ± 7.1 ^c^	12.0 ± 0.5 ^b^	158.5 ± 0.7 ^e^	12.4 ± 0.2 ^i^	3.9 ± 0.1 ^b^	19.3 ± 0.1 ^f^	33.9 ± 0.3 ^g^	-	-	71.7 ± 0.3 ^m^
4B	310.5 ± 8.9 ^bc^	10.7 ± 0.1 ^a^	148.4 ± 3.2 ^d^	12.4 ± 0.3 ^i^	4.2 ± 0.1 ^bc^	18.0 ± 0.6 ^e^	40.0 ± 0.8 ^i^	-	-	74.5 ± 0.1 ^n^
1C	527.3 ± 8.4 ^h^	31.6 ± 0.7 ^k^	268.5 ± 3.9 ^m^	10.8 ± 0.1 ^g^	4.7 ± 0.1 ^d^	23.7 ± 0.2 ^i^	27.1 ± 0.2 ^e^	-	-	41.2 ± 1.3 ^de^
2C	570.2 ± 14.8 ^j^	14.1 ± 0.3 ^d^	176.9 ± 1.5 ^g^	10.7 ± 0.1 ^g^	6.2 ± 0.4 ^f^	24.9 ± 0.2 ^j^	30.9 ± 0.7 ^f^	-	-	59.2 ± 0.6 ^i^
3C	679.2 ± 5.7 ^l^	14.7 ± 0.5 ^de^	131.1 ± 1.5 ^b^	10.9 ± 0.2 ^g^	3.2 ± 0.1 ^b^	17.9 ± 0.1 ^e^	38.0 ± 0.1 ^h^	-	-	64.5 ± 0.7 ^j^
4C	732.9 ± 0.3 ^m^	14.7 ± 0.4 ^de^	113.3 ± 1.5 ^a^	11.8 ± 0.5 ^h^	3.8 ± 0.1 ^b^	17.7 ± 0.6 ^e^	51.1 ± 0.1 ^l^	-	-	69.0 ± 0.2 ^l^
1D	299.7 ± 5.5 ^b^	25.7 ± 0.5 ^i^	237.7 ± 1.6 ^k^	6.8 ± 0.1 ^c^	4.7 ± 0.1 ^d^	30.4 ± 0.2 ^m^	20.5 ± 0.1 ^b^	-	-	16.1 ± 0.1 ^a^
2D	376.8 ± 3.2 ^d^	23.6 ± 0.5 ^h^	197.9 ± 0.6 ^i^	5.9 ± 0.1 ^b^	2.4 ± 0.1 ^a^	16.2 ± 0.4 ^d^	20.7 ± 0.4 ^b^	-	-	30.5 ± 0.1 ^b^
3D	434.8 ± 6.5 ^f^	16.5 ± 0.3 ^f^	184.6 ± 2.5 ^h^	7.4 ± 0.1 ^d^	3.5 ± 0.1 ^b^	12.3 ± 0.1 ^b^	22.7 ± 0.2 ^d^	-	-	52.3 ± 0.1 ^h^
4D	510.6 ± 8.9 ^gh^	14.4 ± 0.1 ^d^	146.6 ± 1.6 ^d^	8.5 ± 0.1 ^e^	3.5 ± 0.1 ^b^	10.3 ± 0.1 ^a^	45.4 ± 0.7 ^h^	-	-	52.2 ± 0.4 ^h^
1E	492.2 ± 6.3 ^g^	16.9 ± 0.2 ^f^	278.9 ± 1.5 ^n^	9.7 ± 0.1 ^f^	4.8 ± 0.1 ^d^	13.4 ± 0.2 ^c^	19.3 ± 0.2 ^a^	-	-	38.1 ± 0.1 ^c^
2E	544.6 ± 2.8 ^i^	13.4 ± 0.4 ^c^	157.1 ± 1.9 ^e^	6.8 ± 0.2 ^c^	5.8 ± 0.3 ^ef^	13.9 ± 0.7 ^c^	33.4 ± 0.1 ^g^	-	-	47.8 ± 0.1 ^f^
3E	627.4 ± 9.3 ^k^	10.2 ± 0.6 ^a^	139.8 ± 1.5 ^c^	5.1 ± 0.1 ^a^	5.2 ± 0.1 ^e^	26.5 ± 0.1 ^k^	41.8 ± 0.1 ^j^	8.4 ± 0.1 ^a^	-	48.8 ± 0.3 ^f^
4E	683.8 ± 8.9 ^l^	10.5 ± 0.2 ^a^	132.3 ± 1.7 ^b^	6.2 ± 0.1 ^b^	5.3 ± 0.1 ^e^	28.0 ± 0.1 ^l^	55.5 ± 0.1 ^m^	12.3 ± 0.7 ^b^	-	53.0 ± 0.4 ^h^

Abbreviations: Me21—2021 vintage Merlot sample prior storage; A—stainless-steel tank; B—wooden barrel with excellent medium toasting; C—wooden barrel with excellent medium plus toasting; D—wooden barrel with excellent medium long toasting; E—wooden barrel with premium medium toasting; 1A–1E—sampling in August 2022; 2A–2E—sampling in November 2022; 3A–3E—sampling in February 2023; 4A–4E—sampling in May 2023.

**Table 5:** Alcohol concentrations in aroma profile of 2020 vintage Merlot and samples obtained during 12-month storage in different vessels. Different superscript letters (a–p) in the same column indicate statistical differences determined by ANOVA and Fisher’s (LSD) test with *p* < 0.05.

Sample	Isoamyl Alcohol (mg/L)	2,3-butanediol (µg/L)	1-hexanol (µg/L)	1-heptanol (µg/L)	Methionol (µg/L)	2-ethyl-1-hexanol (µg/L)	Benzyl Alcohol (µg/L)	1-octanol (µg/L)	2-phenylethanol (mg/L)	Dodecanol (µg/L)
Me20	10.0 ± 0.1 ^a^	664.1 ± 1.6 ^k^	253.6 ± 5.6 ^h^	-	32.2 ± 0.1 ^c^	4.5 ± 0.1 ^a^	28.8 ± 1.3 ^a^	12.5 ± 0.3 ^ab^	3.1 ± 0.1 ^a^	58.1 ± 0.6 ^f^
1a	14.0 ± 0.1 ^e^	638.7 ± 6.8 ^i^	288.2 ± 6.2 ^j^	-	38.9 ± 1.1 ^f^	4.4 ± 0.1 ^a^	39.3 ± 1.5 ^e^	12.3 ± 0.9 ^ab^	3.3 ± 0.1 ^a^	60.6 ± 0.9 ^g^
2a	11.9 ± 0.2 ^b^	636.4 ± 11.5 ^i^	273.6 ± 5.1 ^i^	-	32.2 ± 0.3 ^c^	4.7 ± 0.3 ^a^	34.8 ± 1.6 ^cd^	12.4 ± 0.2 ^ab^	3.9 ± 0.1 ^c^	45.7 ± 0.8 ^c^
3a	11.4 ± 0.2 ^b^	476.6 ± 4.0 ^d^	235.3 ± 2.6 ^f^	-	37.1 ± 0.1 ^f^	4.6 ± 0.3 ^a^	32.4 ± 0.9 ^b^	15.0 ± 0.3 ^c^	3.5 ± 0.1 ^b^	60.7 ± 0.2 ^g^
4a	11.2 ± 0.3 ^b^	250.6 ± 4.9 ^a^	230.3 ± 5.1 ^ef^	-	35.1 ± 1.1 ^e^	5.7 ± 0.1 ^b^	34.1 ± 0.1 ^c^	20.0 ± 0.6 ^g^	3.1 ± 0.1 ^a^	35.0 ± 0.1 ^a^
1b	16.7 ± 0.1 ^g^	429.5 ± 8.2 ^b^	247.7 ± 0.8 ^gh^	-	29.9 ± 0.1 ^b^	45.8 ± 1.1 ^m^	75.4 ± 2.5 ^j^	23.7 ± 0.1 ^i^	3.9 ± 0.1 ^c^	72.6 ± 1.5 ^i^
2b	15.6 ± 0.2 ^f^	492.6 ± 9.9 ^e^	243.6 ± 4.2 ^g^	-	32.2 ± 0.2 ^c^	23.1 ± 0.8 ^j^	43.2 ± 0.5 ^g^	21.5 ± 0.1 ^h^	3.8 ± 0.1 ^c^	97.5 ± 1.0 ^n^
3b	11.5 ± 0.2 ^b^	444.4 ± 1.0 ^c^	290.0 ± 0.6 ^j^	-	33.1 ± 0.5 ^d^	17.6 ± 0.1 ^h^	34.8 ± 0.3 ^c^	24.2 ± 0.5 ^i^	4.0 ± 0.1 ^cd^	111.7 ± 1.6 ^p^
4b	10.3 ± 0.2 ^a^	558.1 ± 4.3 ^g^	303.2 ± 0.6 ^k^	-	37.8 ± 0.1 ^f^	9.2 ± 0.1 ^d^	31.8 ± 0.3 ^b^	18.8 ± 0.1 ^f^	4.1 ± 0.1 ^d^	82.3 ± 0.5 ^j^
1c	13.8 ± 0.2 ^de^	568.6 ± 2.0 ^g^	144.3 ± 0.6 ^a^	-	38.6 ± 1.3 ^b^	21.8 ± 0.9 ^ij^	41.8 ± 0.3 ^f^	14.7 ± 0.4 ^c^	4.1 ± 0.1 ^d^	67.6 ± 1.2 ^h^
2c	14.2 ± 0.1 ^e^	581.8 ± 5.3 ^h^	154.3 ± 2.7 ^b^	-	31.2 ± 0.3 ^c^	16.1 ± 0.4 ^g^	49.7 ± 0.4 ^h^	17.9 ± 0.6 ^e^	4.2 ± 0.1 ^d^	85.6 ± 0.2 ^k^
3c	13.4 ± 0.1 ^d^	576.8 ± 8.5 ^h^	156.3 ± 0.6 ^b^	-	45.9 ± 3.1 ^g^	16.4 ± 0.1 ^g^	31.5 ± 0.6 ^b^	13.0 ± 0.6 ^ab^	3.1 ± 0.1 ^a^	92.8 ± 0.8 ^l^
4c	14.2 ± 0.3 ^e^	654.6 ± 2.3 ^j^	162.2 ± 0.7 ^c^	-	45.0 ± 0.4 ^g^	7.3 ± 0.1 ^c^	36.1 ± 0.2 ^d^	13.8 ± 0.1 ^c^	3.4 ± 0.1 ^b^	106.1 ± 0.7 ^o^
1d	11.8 ± 0.1 ^b^	427.5 ± 6.4 ^b^	221.4 ± 2.0 ^e^	-	29.0 ± 0.5 ^b^	26.0 ± 0.8 ^k^	43.8 ± 0.9 ^g^	17.6 ± 0.1 ^e^	3.7 ± 0.1 ^bc^	55.3 ± 1.1 ^e^
2d	14.1 ± 0.1 ^e^	430.0 ± 4.0 ^b^	271.5 ± 5.0 ^i^	-	28.7 ± 0.2 ^b^	13.2 ± 0.1 ^f^	50.3 ± 0.1 ^h^	12.7 ± 0.3 ^ab^	3.7 ± 0.1 ^bc^	86.9 ± 1.4 ^k^
3d	15.5 ± 0.1 ^f^	645.9 ± 1.6 ^i^	325.2 ± 2.1 ^l^	-	26.6 ± 0.2 ^a^	11.0 ± 0.1 ^e^	39.9 ± 0.8 ^e^	13.3 ± 0.1 ^b^	4.0 ± 0.1 ^cd^	92.5 ± 0.5 ^l^
4d	16.4 ± 0.1 ^g^	664.2 ± 3.7 ^k^	325.5 ± 1.4 ^l^	-	26.7 ± 0.8 ^a^	9.8 ± 0.1 ^d^	41.1 ± 0.4 ^f^	13.2 ± 0.1 ^b^	3.3 ± 0.1 ^ab^	94.8 ± 0.1 ^m^
1e	11.7 ± 0.1 ^b^	452.8 ± 9.1 ^c^	200.3 ± 0.6 ^d^	-	33.9 ± 1.9 ^cde^	30.3 ± 0.4 ^i^	44.2 ± 1.1 ^g^	12.3 ± 0.2 ^a^	4.2 ± 0.2 ^d^	42.1 ± 1.3 ^b^
2e	12.1 ± 0.1 ^c^	536.3 ± 1.3 ^f^	279.8 ± 4.3 ^i^	-	34.7 ± 1.2 ^de^	26.5 ± 0.1 ^k^	53.9 ± 0.1 ^i^	16.5 ± 0.6 ^d^	4.1 ± 0.2 ^d^	42.5 ± 0.2 ^b^
3e	15.8 ± 0.1 ^f^	477.2 ± 0.5 ^d^	324.6 ± 1.6 ^l^	-	26.2 ± 0.2 ^a^	30.0 ± 0.1 ^l^	43.4 ± 0.2 ^g^	16.2 ± 0.3 ^d^	4.0 ± 0.1 ^cd^	49.5 ± 0.1 ^d^
4e	15.4 ± 0.1 ^d^	461.0 ± 7.3 ^c^	356.1 ± 1.4 ^m^	-	28.2 ± 1.6 ^b^	27.2 ± 0.2 ^k^	43.7 ± 0.6 ^g^	15.9 ± 0.3 ^d^	3.9 ± 0.1 ^c^	55.7 ± 0.5 ^e^

Abbreviations: Me20—2020 vintage Merlot sample prior storage; a—stainless-steel tank; b—wooden barrel with excellent medium toasting; c—wooden barrel with excellent medium plus toasting; d—wooden barrel with excellent medium long toasting; e—wooden barrel with premium medium toasting; 1a–1e—sampling in June 2021; 2a–2e—sampling in September 2021; 3a–3e—sampling in December 2021; 4a–4e—sampling in March 2022.

**Table 6:** Alcohol concentrations in aroma profile of 2021 vintage Merlot and samples obtained during 12-month storage in different vessels. Different superscript letters (a–q) in the same column indicate statistical differences determined by ANOVA and Fisher’s (LSD) test with *p* < 0.05.

Sample	Isoamyl Alcohol (mg/L)	2,3-butanediol (µg/L)	1-hexanol (µg/L)	1-heptanol (µg/L)	Methionol (µg/L)	2-ethyl-1-hexanol (µg/L)	Benzyl Alcohol (µg/L)	1-octanol (µg/L)	2-phenylethanol (mg/L)	Dodecanol (µg/L)
Me21	6.5 ± 0.2 ^a^	416.4 ± 4.3 ^e^	125.0 ± 2.4 ^e^	6.2 ± 0.1 ^c^	40.7 ± 0.9 ^l^	4.5 ± 0.2 ^a^	11.2 ± 0.4 ^e^	19.6 ± 0.1 ^e^	2.5 ± 0.1 ^ab^	46.7 ± 1.2 ^ef^
1A	7.8 ± 0.1 ^b^	692.9 ± 4.2 ^k^	273.8 ± 10.4 ^l^	7.8 ± 0.1 ^d^	29.0 ± 0.2 ^h^	4.6 ± 0.2 ^a^	11.7 ± 0.3 ^e^	22.0 ± 0.2 ^f^	2.9 ± 0.1 ^c^	56.0 ± 0.4 ^i^
2A	7.2 ± 0.2 ^b^	686.7 ± 6.6 ^k^	211.2 ± 8.7 ^i^	5.7 ± 0.1 ^b^	23.6 ± 0.5 ^e^	6.4 ± 0.2 ^c^	7.0 ± 0.3 ^a^	21.6 ± 0.6 ^f^	3.0 ± 0.1 ^c^	46.9 ± 0.9 ^e^
3A	6.9 ± 0.1 ^ab^	520.2 ± 6.8 ^i^	130.4 ± 0.9 ^f^	10.7 ± 0.1 ^f^	27.0 ± 0.1 ^g^	5.9 ± 0.2 ^b^	6.9 ± 0.2 ^a^	24.1 ± 0.2 ^h^	2.9 ± 0.1 ^c^	55.4 ± 1.9 ^hi^
4A	6.8 ± 0.1 ^b^	450.8 ± 2.3 ^f^	126.5 ± 2.4 ^e^	10.8 ± 0.1 ^f^	22.2 ± 0.2 ^d^	6.3 ± 0.2 ^c^	7.6 ± 0.1 ^a^	27.2 ± 0.2 ^j^	2.6 ± 0.1 ^b^	46.2 ± 0.4 ^e^
1B	7.4 ± 0.1 ^b^	374.0 ± 3.1 ^b^	126.4 ± 2.9 ^e^	4.9 ± 0.1 ^a^	19.0 ± 0.2 ^b^	38.9 ± 0.1 ^i^	13.0 ± 0.1 ^g^	26.1 ± 0.6 ^j^	2.9 ± 0.1 ^c^	59.6 ± 0.2 ^k^
2B	7.0 ± 0.1 ^b^	468.9 ± 7.3 ^g^	123.8 ± 0.2 ^e^	4.6 ± 0.1 ^a^	20.7 ± 0.1 ^c^	23.4 ± 0.3 ^h^	10.7 ± 0.1 ^d^	18.6 ± 0.2 ^d^	2.9 ± 0.1 ^c^	68.9 ± 0.1 ^m^
3B	6.8 ± 0.1 ^ab^	490.6 ± 6.3 ^h^	157.6 ± 0.5 ^g^	6.7 ± 0.1 ^c^	26.5 ± 0.2 ^f^	19.9 ± 0.1 ^f^	7.8 ± 0.4 ^a^	18.6 ± 0.1 ^d^	2.9 ± 0.1 ^c^	71.0 ± 0.2 ^n^
4B	6.5 ± 0.1 ^a^	528.8 ± 3.1 ^i^	159.2 ± 0.1 ^g^	6.4 ± 0.1 ^c^	31.9 ± 0.4 ^i^	18.4 ± 0.1 ^e^	7.1 ± 0.2 ^a^	17.0 ± 0.1 ^c^	3.2 ± 0.1 ^d^	67.0 ± 0.4 ^l^
1C	8.5 ± 0.2 ^c^	444.8 ± 0.9 ^f^	113.9 ± 1.7 ^d^	6.2 ± 0.1 ^c^	36.3 ± 0.1 ^k^	5.9 ± 0.1 ^b^	14.1 ± 0.1 ^h^	23.0 ± 0.4 ^g^	3.1 ± 0.1 ^c^	73.2 ± 0.6 ^o^
2C	11.7 ± 0.3 ^ef^	442.2 ± 5.0 ^f^	211.9 ± 6.4 ^i^	5.9 ± 0.1 ^bc^	34.6 ± 0.2 ^j^	5.6 ± 0.2 ^c^	9.4 ± 0.2 ^c^	25.1 ± 0.2 ^i^	3.1 ± 0.1 ^c^	92.6 ± 0.4 ^p^
3C	12.0 ± 0.1 ^f^	642.1 ± 1.6 ^j^	225.5 ± 2.0 ^j^	11.8 ± 0.1 ^g^	42.3 ± 0.6 ^m^	5.2 ± 0.1 ^b^	8.7 ± 0.4 ^b^	22.1 ± 0.3 ^f^	2.8 ± 0.1 ^bc^	91.8 ± 1.3 ^p^
4C	14.7 ± 0.2 ^g^	742.7 ± 5.4 ^l^	256.5 ± 0.2 ^k^	12.1 ± 0.1 ^g^	42.5 ± 1.2 ^lm^	5.3 ± 0.1 ^b^	10.7 ± 0.2 ^d^	24.2 ± 0.1 ^h^	3.0 ± 0.1 ^c^	100.3 ± 1.3 ^q^
1D	9.8 ± 0.1 ^d^	231.1 ± 1.5 ^a^	73.3 ± 1.9 ^a^	7.1 ± 0.1 ^d^	28.3 ± 1.0 ^gh^	22.5 ± 0.2 ^b^	8.1 ± 0.2 ^b^	16.6 ± 0.3 ^b^	2.6 ± 0.1 ^b^	34.2 ± 0.5 ^a^
2D	10.1 ± 0.1 ^d^	230.0 ± 4.9 ^a^	82.4 ± 0.3 ^b^	9.3 ± 0.1 ^e^	26.5 ± 0.4 ^fg^	12.3 ± 0.3 ^g^	12.8 ± 0.2 ^fg^	16.3 ± 0.3 ^b^	2.6 ± 0.1 ^b^	43.8 ± 0.2 ^c^
3D	10.8 ± 0.1 ^e^	1096.0 ± 5.8 ^m^	112.8 ± 2.4 ^d^	12.1 ± 0.1 ^g^	14.9 ± 0.7 ^a^	6.3 ± 0.1 ^d^	8.0 ± 0.1 ^b^	17.4 ± 0.1 ^c^	2.6 ± 0.1 ^b^	48.6 ± 0.6 ^f^
4D	16.7 ± 0.2 ^h^	1162.6 ± 3.2 ^n^	113.5 ± 0.1 ^d^	12.3 ± 0.1 ^gh^	15.1 ± 0.1 ^a^	5.5 ± 0.1 ^c^	8.1 ± 0.1 ^b^	18.6 ± 0.2 ^d^	2.4 ± 0.1 ^a^	53.4 ± 0.2 ^n^
1E	10.0 ± 0.1 ^d^	386.5 ± 1.8 ^c^	95.9 ± 0.1 ^c^	10.1 ± 0.1 ^f^	45.7 ± 0.3 ^n^	67.0 ± 0.5 ^b^	12.3 ± 0.1 ^f^	29.4 ± 0.6 ^k^	2.9 ± 0.1 ^c^	40.5 ± 0.2 ^b^
2E	11.5 ± 0.1 ^e^	473.5 ± 0.6 ^g^	114.7 ± 0.5 ^d^	12.7 ± 0.1 ^h^	25.1 ± 1.1 ^ef^	67.6 ± 0.4 ^k^	14.7 ± 0.1 ^h^	29.4 ± 0.1 ^k^	3.0 ± 0.1 ^c^	44.7 ± 0.2 ^d^
3E	12.7 ± 0.1 ^f^	412.0 ± 1.2 ^e^	179.8 ± 0.9 ^h^	5.8 ± 0.1 ^b^	22.3 ± 0.2 ^d^	64.1 ± 0.5 ^k^	10.8 ± 0.4 ^d^	13.5 ± 0.2 ^a^	2.8 ± 0.1 ^bc^	50.7 ± 0.1 ^g^
4E	12.4 ± 0.1 ^f^	398.9 ± 0.9 ^d^	212.8 ± 0.1 ^i^	5.7 ± 0.1 ^b^	22.0 ± 0.1 ^d^	64.0 ± 0.6 ^j^	10.8 ± 0.3 ^d^	13.1 ± 0.3 ^a^	2.6 ± 0.1 ^b^	57.2 ± 0.4 ^j^

Abbreviations: Me21—2021 vintage Merlot sample prior storage; A—stainless-steel tank; B—wooden barrel with excellent medium toasting; C—wooden barrel with excellent medium plus toasting; D—wooden barrel with excellent medium long toasting; E—wooden barrel with premium medium toasting; 1A–1E—sampling in August 2022; 2A–2E—sampling in November 2022; 3A–3E—sampling in February 2023; 4A–4E—sampling in May 2023.

**Table 7:** Concentrations of carbonyl compounds and terpenes (µg/L) in aroma profile of 2020 vintage Merlot and samples obtained during 12-month storage in different vessels. Different superscript letters (a–m) in the same column indicate statistical differences determined by ANOVA and Fisher’s (LSD) test with *p* < 0.05.

Sample	Carbonyl Compounds				Terpenes				
	4-propylbenzaldehyde	Geranyl Acetone	Lily Aldehyde	Hexyl Cinnamaldehyde	Linalool	Hotrienol	β-citronellol	Eugenol	β-damascenone
Me20	26.8 ± 0.3 ^g^	14.9 ± 0.1 ^g^	-	7.6 ± 0.1 ^e^	17.3 ± 0.2 ^b^	32.7 ± 1.1 ^c^	5.9 ± 0.1 ^c^	-	5.3 ± 0.2 ^b^
1a	9.4 ± 0.5 ^e^	11.4 ± 0.1 ^d^	4.7 ± 0.1 ^b^	8.7 ± 0.1 ^f^	37.3 ± 1.2 ^hi^	66.9 ± 0.1 ^k^	6.9 ± 0.2 ^d^	-	8.4 ± 0.3 ^ef^
2a	8.1 ± 0.3 ^cd^	11.9 ± 0.1 ^d^	3.2 ± 0.1 ^a^	4.9 ± 0.1 ^bc^	46.1 ± 2.0 ^lm^	85.3 ± 1.0 ^m^	5.4 ± 0.1 ^b^	-	6.5 ± 0.2 ^c^
3a	5.5 ± 0.1 ^a^	11.7 ± 0.1 ^d^	3.8 ± 0.1 ^a^	4.3 ± 0.1 ^b^	22.5 ± 0.1 ^c^	37.0 ± 0.4 ^e^	6.0 ± 0.1 ^c^	-	7.8 ± 0.1 ^d^
4a	5.7 ± 0.1 ^a^	8.9 ± 0.1 ^b^	3.2 ± 0.1 ^a^	3.3 ± 0.1 ^a^	31.9 ± 1.0 ^f^	32.0 ± 0.9 ^c^	5.1 ± 0.1 ^b^	-	4.0 ± 0.1 ^a^
1b	10.5 ± 0.4 ^f^	13.6 ± 0.1 ^f^	7.8 ± 0.1 ^e^	9.6 ± 0.1 ^g^	44.6 ± 0.5 ^l^	59.6 ± 0.1 ^j^	7.9 ± 0.1 ^f^	3.6 ± 0.2 ^a^	13.2 ± 0.7 ^j^
2b	9.4 ± 0.5 ^e^	14.6 ± 0.1 ^g^	10.3 ± 0.1 ^h^	8.1 ± 0.1 ^f^	40.6 ± 0.1 ^j^	55.1 ± 0.7 ^i^	7.8 ± 0.1 ^f^	5.6 ± 0.2 ^c^	11.5 ± 0.3 ^hi^
3b	9.2 ± 0.2 ^e^	11.8 ± 0.1 ^d^	12.9 ± 0.1 ^i^	7.5 ± 0.1 ^e^	41.8 ± 0.2 ^k^	61.3 ± 1.2 ^j^	7.1 ± 0.3 ^e^	7.4 ± 0.1 ^e^	7.5 ± 0.1 ^d^
4b	7.5 ± 0.2 ^bc^	12.6 ± 0.1 ^e^	8.8 ± 0.1 ^f^	7.2 ± 0.1 ^e^	38.0 ± 0.3 ^i^	41.2 ± 0.2 ^f^	5.3 ± 0.1 ^b^	7.4 ± 0.2 ^e^	6.6 ± 0.1 ^c^
1c	9.0 ± 0.1 ^e^	10.6 ± 0.2 ^c^	4.7 ± 0.1 ^b^	5.3 ± 0.1 ^c^	36.4 ± 0.6 ^h^	29.8 ± 0.6 ^b^	6.4 ± 0.1 ^d^	4.5 ± 0.4 ^b^	11.0 ± 0.1 ^h^
2c	9.5 ± 0.1 ^e^	11.7 ± 0.1 ^d^	6.1 ± 0.1 ^d^	8.2 ± 0.1 ^f^	43.9 ± 1.5 ^l^	61.3 ± 1.0 ^j^	6.6 ± 0.1 ^d^	7.1 ± 0.1 ^e^	15.1 ± 0.1 ^k^
3c	9.2 ± 0.2 ^e^	14.8 ± 0.1 ^g^	6.9 ± 0.1 ^d^	8.3 ± 0.1 ^f^	34.2 ± 0.5 ^g^	67.6 ± 2.1 ^k^	4.8 ± 0.1 ^a^	7.7 ± 0.1 ^e^	7.5 ± 0.1 ^d^
4c	9.0 ± 0.1 ^e^	14.8 ± 0.1 ^g^	9.1 ± 0.1 ^g^	9.9 ± 0.1 ^g^	29.2 ± 0.2 ^e^	73.9 ± 0.1 ^l^	5.9 ± 0.1 ^c^	8.4 ± 0.2 ^f^	7.6 ± 0.1 ^d^
1d	7.6 ± 0.1 ^c^	10.6 ± 0.1 ^c^	6.5 ± 0.1 ^d^	6.2 ± 0.1 ^d^	47.6 ± 0.2 ^m^	27.6 ± 0.1 ^a^	8.9 ± 0.2 ^g^	3.1 ± 0.1 ^a^	12.0 ± 0.3 ^i^
2d	7.2 ± 0.1 ^b^	9.1 ± 0.1 ^b^	4.0 ± 0.1 ^ab^	4.6 ± 0.1 ^b^	28.1 ± 0.1 ^e^	64.5 ± 1.4 ^jk^	6.7 ± 0.2 ^d^	5.1 ± 0.1 ^c^	6.8 ± 0.1 ^c^
3d	8.0 ± 0.1 ^c^	9.0 ± 0.1 ^b^	4.4 ± 0.1 ^b^	4.1 ± 0.1 ^b^	26.8 ± 0.1 ^d^	62.2 ± 2.0 ^jk^	5.9 ± 0.1 ^c^	6.6 ± 0.1 ^d^	6.6 ± 0.1 ^c^
4d	8.6 ± 0.1 ^d^	8.2 ± 0.1 ^a^	3.7 ± 0.1 ^a^	4.1 ± 0.1 ^b^	22.0 ± 0.4 ^c^	59.8 ± 0.2 ^j^	4.8 ± 0.1 ^a^	6.8 ± 0.1 ^d^	5.2 ± 0.1 ^b^
1e	7.6 ± 0.1 ^bc^	-	5.2 ± 0.1 ^c^	5.1 ± 0.1 ^c^	21.8 ± 0.2 ^c^	45.8 ± 0.3 ^g^	4.7 ± 0.1 ^a^	5.3 ± 0.3 ^c^	8.7 ± 0.1 ^f^
2e	8.6 ± 0.2 ^d^	-	5.1 ± 0.1 ^c^	7.6 ± 0.1 ^e^	17.9 ± 0.1 ^b^	49.9 ± 0.1 ^h^	5.9 ± 0.1 ^c^	8.3 ± 0.2 ^f^	10.3 ± 0.2 ^g^
3e	7.2 ± 0.1 ^b^	-	4.8 ± 0.1 ^bc^	6.2 ± 0.1 ^d^	17.2 ± 0.2 ^b^	34.5 ± 0.4 ^d^	7.2 ± 0.1 ^e^	10.9 ± 0.3 ^g^	8.6 ± 0.2 ^f^
4e	7.8 ± 0.1 ^c^	-	4.6 ± 0.1 ^b^	6.6 ± 0.1 ^d^	16.5 ± 0.3 ^a^	34.6 ± 1.1 ^cd^	7.3 ± 0.1 ^e^	11.1 ± 0.1 ^g^	8.0 ± 0.1 ^e^

Abbreviations: Me20—2020 vintage Merlot sample prior storage; a—stainless-steel tank; b—wooden barrel with excellent medium toasting; c—wooden barrel with excellent medium plus toasting; d—wooden barrel with excellent medium long toasting; e—wooden barrel with premium medium toasting; 1a–1e—sampling in June 2021; 2a–2e—sampling in September 2021; 3a–3e—sampling in December 2021; 4a–4e—sampling in March 2022.

**Table 8:** Concentrations of carbonyl compounds and terpenes (µg/L) in aroma profile of 2021 vintage Merlot and samples obtained during 12-month storage in different vessels. Different superscript letters (a–k) in the same column indicate statistical differences determined by ANOVA and Fisher’s (LSD) test with *p* < 0.05.

Sample	Carbonyl Compounds				Terpenes				
	4-propylbenzaldehyde	Geranyl Acetone	Lily Aldehyde	Hexyl Cinnamaldehyde	Linalool	Hotrienol	β-citronellol	Eugenol	β-damascenone
Me21	12.5 ± 0.1 ^h^	9.5 ± 0.1 ^e^	7.1 ± 0.1 ^c^	7.4 ± 0.1 ^d^	12.4 ± 0.2 ^d^	7.7 ± 0.1 ^b^	10.6 ± 0.3 ^e^	-	7.3 ± 0.2 ^d^
1A	5.4 ± 0.2 ^d^	7.6 ± 0.1 ^c^	6.4 ± 0.1 ^b^	8.9 ± 0.1 ^e^	12.2 ± 0.2 ^d^	6.8 ± 0.1 ^a^	10.8 ± 0.3 ^e^	-	7.7 ± 0.1 ^c^
2A	4.6 ± 0.1 ^c^	7.5 ± 0.1 ^c^	5.1 ± 0.1 ^a^	6.7 ± 0.1 ^c^	24.4 ± 0.1 ^j^	12.6 ± 0.1 ^f^	7.3 ± 0.1 ^c^	-	6.2 ± 0.1 ^bc^
3A	3.3 ± 0.1 ^b^	7.6 ± 0.1 ^c^	7.8 ± 0.1 ^c^	6.7 ± 0.1 ^c^	16.6 ± 0.2 ^f^	10.2 ± 0.1 ^d^	8.2 ± 0.1 ^d^	-	7.3 ± 0.1 ^d^
4A	3.6 ± 0.1 ^b^	6.7 ± 0.1 ^b^	6.7 ± 0.1 ^b^	4.5 ± 0.1 ^a^	16.7 ± 0.2 ^f^	8.3 ± 0.1 ^c^	7.3 ± 0.1 ^c^	-	5.2 ± 0.1 ^a^
1B	5.4 ± 0.1 ^d^	8.7 ± 0.1 ^d^	8.6 ± 0.1 ^d^	6.2 ± 0.1 ^c^	19.5 ± 0.1 ^h^	10.4 ± 0.1 ^d^	11.7 ± 0.1 ^f^	3.3 ± 0.2 ^bc^	8.8 ± 0.1 ^f^
2B	4.9 ± 0.1 ^c^	8.7 ± 0.1 ^d^	10.7 ± 0.1 ^e^	5.4 ± 0.1 ^b^	15.5 ± 0.2 ^e^	10.0 ± 0.1 ^d^	10.8 ± 0.1 ^e^	3.7 ± 0.1 ^c^	7.3 ± 0.1 ^d^
3B	4.8 ± 0.1 ^c^	7.2 ± 0.1 ^c^	13.6 ± 0.1 ^e^	4.7 ± 0.1 ^a^	16.5 ± 0.1 ^f^	11.1 ± 0.1 ^e^	7.4 ± 0.1 ^c^	4.3 ± 0.1 ^d^	6.3 ± 0.1 ^bc^
4B	3.2 ± 0.1 ^b^	8.9 ± 0.1 ^d^	13.0 ± 0.1 ^e^	4.9 ± 0.1 ^ab^	15.6 ± 0.2 ^e^	8.8 ± 0.1 ^c^	6.6 ± 0.1 ^b^	4.4 ± 0.1 ^d^	5.8 ± 0.1 ^b^
1C	10.8 ± 0.4 ^g^	7.7 ± 0.1 ^c^	15.4 ± 0.2 ^g^	6.8 ± 0.1 ^cd^	21.1 ± 0.1 ^i^	10.3 ± 0.1 ^d^	10.5 ± 0.1 ^e^	2.3 ± 0.1 ^a^	14.8 ± 0.2 ^i^
2C	16.1 ± 0.8 ^i^	9.3 ± 0.1 ^e^	19.2 ± 0.2 ^h^	8.9 ± 0.1 ^e^	21.4 ± 0.2 ^i^	13.7 ± 0.1 ^g^	10.2 ± 0.3 ^e^	3.0 ± 0.1 ^b^	17.9 ± 0.2 ^j^
3C	17.5 ± 0.1 ^j^	11.7 ± 0.1 ^g^	21.3 ± 0.3 ^i^	9.2 ± 0.1 ^e^	19.4 ± 0.1 ^h^	16.8 ± 0.1 ^g^	6.8 ± 0.1 ^b^	3.6 ± 0.1 ^c^	9.6 ± 0.1 ^g^
4C	19.4 ± 0.1 ^k^	11.7 ± 0.1 ^g^	23.3 ± 0.3 ^j^	10.2 ± 0.1 ^f^	18.5 ± 0.3 ^g^	17.7 ± 0.1 ^h^	6.7 ± 0.1 ^b^	4.6 ± 0.1 ^d^	9.3 ± 0.1 ^g^
1D	2.6 ± 0.2 ^a^	8.2 ± 0.1 ^d^	8.7 ± 0.1 ^d^	6.3 ± 0.1 ^c^	12.9 ± 0.1 ^d^	7.4 ± 0.1 ^b^	10.6 ± 0.1 ^e^	-	10.8 ± 0.2 ^h^
2D	2.8 ± 0.1 ^a^	7.8 ± 0.1 ^c^	5.7 ± 0.1 ^a^	6.8 ± 0.1 ^cd^	8.1 ± 0.1 ^c^	12.2 ± 0.1 ^f^	6.9 ± 0.1 ^b^	2.6 ± 0.1 ^a^	7.1 ± 0.1 ^d^
3D	5.5 ± 0.1 ^d^	7.9 ± 0.1 ^c^	6.4 ± 0.1 ^b^	6.0 ± 0.1 ^c^	7.3 ± 0.1 ^b^	12.1 ± 0.1 ^f^	4.6 ± 0.1 ^a^	2.6 ± 0.1 ^a^	5.2 ± 0.1 ^a^
4D	6.4 ± 0.1 ^e^	7.9 ± 0.1 ^c^	5.2 ± 0.1 ^a^	6.1 ± 0.1 ^c^	5.6 ± 0.1 ^a^	10.5 ± 0.1 ^d^	4.7 ± 0.1 ^a^	3.5 ± 0.2 ^bc^	4.9 ± 0.1 ^a^
1E	5.4 ± 0.1 ^d^	10.4 ± 0.1 ^f^	13.7 ± 0.1 ^e^	6.0 ± 0.1 ^c^	16.9 ± 0.1 ^f^	10.1 ± 0.1 ^d^	7.1 ± 0.1 ^c^	3.6 ± 0.1 ^c^	7.8 ± 0.1 ^e^
2E	6.8 ± 0.1 ^e^	7.8 ± 0.1 ^c^	14.7 ± 0.1 ^f^	5.4 ± 0.1 ^b^	18.4 ± 0.1 ^g^	13.8 ± 0.1 ^g^	7.0 ± 0.1 ^c^	4.4 ± 0.1 ^d^	9.1 ± 0.1 ^fg^
3E	6.5 ± 0.1 ^e^	5.5 ± 0.1 ^a^	7.2 ± 0.1 ^c^	4.4 ± 0.1 ^a^	12.5 ± 0.1 ^d^	8.6 ± 0.1 ^c^	7.3 ± 0.1 ^c^	4.5 ± 0.1 ^d^	7.8 ± 0.1 ^e^
4E	7.3 ± 0.1 ^g^	5.6 ± 0.1 ^a^	7.2 ± 0.1 ^c^	4.5 ± 0.1 ^a^	12.7 ± 0.1 ^d^	8.5 ± 0.1 ^c^	7.3 ± 0.1 ^c^	4.5 ± 0.2 ^d^	6.5 ± 0.1 ^c^

Abbreviations: Me21—2021 vintage Merlot sample prior storage; A—stainless-steel tank; B—wooden barrel with excellent medium toasting; C—wooden barrel with excellent medium plus toasting; D—wooden barrel with excellent medium long toasting; E—wooden barrel with premium medium toasting; 1A–1E—sampling in August 2022; 2A–2E—sampling in November 2022; 3A–3E—sampling in February 2023; 4A–4E—sampling in May 2023.

**Table 9:** Ester concentrations (µg/L) in aroma profile of 2020 vintage Merlot and samples obtained during 12-month storage in different vessels. Different superscript letters (a–p) in the same column indicate statistical differences determined by ANOVA and Fisher’s (LSD) test with *p* < 0.05.

Sample	Ethyl Hexanoate	Ethyl 4-hydroxybutanoate	Diethyl Succinate	Ethyl Octanoate	Ethyl Hydrogen Succinate	Phenethyl Acetate	Ethyl Decanoate	Ethyl Cinnamate	Ethyl Vanillate	Ethyl Laurate
Me20	65.5 ± 2.5 ^c^	13.0 ± 0.5 ^e^	1039.4 ± 20.1 ^g^	195.9 ± 2.7 ^e^	-	39.7 ± 0.5 ^c^	18.2 ± 0.1 ^b^	5.5 ± 0.2 ^c^	6.1 ± 0.1 ^b^	6.0 ± 0.1 ^g^
1a	71.5 ± 0.1 ^cd^	20.4 ± 0.1 ^g^	1445.1 ± 31.9 ^def^	174.9 ± 1.2 ^c^	-	42.7 ± 0.5 ^e^	21.3 ± 0.9 ^de^	5.5 ± 0.1 ^c^	5.4 ± 0.1 ^a^	4.7 ± 0.1 ^d^
2a	77.8 ± 1.7 ^f^	12.6 ± 0.5 ^e^	1607.5 ± 23.7 ^ij^	192.2 ± 2.3 ^e^	-	35.0 ± 1.5 ^a^	20.9 ± 0.8 ^d^	5.5 ± 0.1 ^c^	5.9 ± 0.1 ^ab^	3.4 ± 0.1 ^c^
3a	84.0 ± 0.1 ^h^	9.7 ± 0.7 ^c^	1529.2 ± 2.0 ^g^	218.4 ± 0.5 ^g^	-	38.0 ± 0.1 ^b^	21.2 ± 1.0 ^de^	5.6 ± 0.2 ^c^	5.5 ± 0.1 ^a^	2.8 ± 0.1 ^ab^
4a	82.1 ± 0.2 ^g^	7.0 ± 0.1 ^b^	1478.3 ± 24.4 ^def^	210.7 ± 3.0 ^f^	-	37.9 ± 0.3 ^b^	17.0 ± 0.1 ^a^	3.6 ± 0.1 ^a^	5.4 ± 0.1 ^a^	2.5 ± 0.1 ^a^
1b	104.3 ± 1.1 ^i^	10.7 ± 0.5 ^cd^	1590.2 ± 5.5 ^i^	226.0 ± 0.1 ^h^	84.9 ± 1.5 ^f^	53.2 ± 0.2 ^j^	31.5 ± 0.4 ^k^	8.0 ± 0.1 ^f^	9.0 ± 0.1 ^g^	4.1 ± 0.1 ^c^
2b	113.3 ± 0.1 ^j^	11.3 ± 0.1 ^d^	1513.5 ± 16.5 ^g^	206.3 ± 4.2 ^f^	130.4 ± 0.9 ^i^	49.6 ± 0.1 ^h^	29.7 ± 0.1 ^j^	6.1 ± 0.1 ^d^	12.0 ± 0.2 ^i^	7.2 ± 0.1 ^i^
3b	112.5 ± 1.0 ^j^	11.7 ± 0.1 ^d^	1535.4 ± 4.5 ^h^	261.0 ± 1.2 ^j^	106.0 ± 1.4 ^g^	51.4 ± 0.1 ^i^	18.1 ± 0.1 ^b^	6.0 ± 0.1 ^d^	8.7 ± 0.2 ^fg^	4.6 ± 0.1 ^d^
4b	81.4 ± 0.3 ^g^	12.4 ± 0.5 ^e^	1639.0 ± 15.2 ^j^	150.0 ± 0.2 ^a^	60.6 ± 0.3 ^d^	49.1 ± 0.3 ^h^	18.4 ± 0.1 ^b^	4.7 ± 0.1 ^b^	7.4 ± 0.1 ^d^	4.5 ± 0.1 ^d^
1c	105.6 ± 1.0 ^k^	12.9 ± 0.7 ^e^	1647.4 ± 19.1 ^i^	205.0 ± 2.9 ^f^	77.8 ± 1.4 ^e^	42.3 ± 0.9 ^e^	28.9 ± 0.6 ^j^	6.6 ± 0.1 ^e^	9.6 ± 0.1 ^gh^	3.0 ± 0.1 ^b^
2c	56.9 ± 0.6 ^b^	13.9 ± 0.2 ^e^	1731.2 ± 14.9 ^l^	255.2 ± 1.4 ^i^	135.4 ± 0.6 ^j^	50.4 ± 0.1 ^h^	23.2 ± 0.1 ^f^	8.6 ± 0.2 ^g^	11.8 ± 0.2 ^i^	2.4 ± 0.1 ^a^
3c	69.4 ± 1.3 ^c^	7.2 ± 0.2 ^b^	1338.3 ± 15.2 ^b^	205.0 ± 1.9 ^f^	53.7 ± 0.1 ^c^	43.8 ± 0.3 ^e^	21.0 ± 0.1 ^d^	5.6 ± 0.1 ^c^	9.7 ± 0.1 ^h^	2.5 ± 0.1 ^a^
4c	98.9 ± 0.4 ^h^	10.3 ± 0.1 ^c^	1442.8 ± 1.7 ^d^	206.0 ± 0.2 ^f^	32.7 ± 0.3 ^a^	44.9 ± 0.2 ^f^	25.3 ± 0.4 ^g^	5.8 ± 0.1 ^cd^	8.9 ± 0.2 ^fg^	2.5 ± 0.1 ^a^
1d	96.7 ± 0.8 ^h^	13.8 ± 0.5 ^ef^	1381.5 ± 6.5 ^c^	178.0 ± 0.1 ^c^	138.0 ± 1.4 ^j^	41.3 ± 0.4 ^d^	27.1 ± 0.2 ^i^	5.9 ± 0.1 ^cd^	6.7 ± 0.1 ^c^	4.1 ± 0.1 ^c^
2d	73.7 ± 0.7 ^e^	14.2 ± 0.1 ^f^	1482.8 ± 3.2 ^f^	184.2 ± 2.7 ^d^	157.8 ± 0.9 ^k^	37.4 ± 0.2 ^b^	17.2 ± 0.7 ^a^	4.9 ± 0.1 ^b^	6.9 ± 0.1 ^cd^	6.4 ± 0.1 ^h^
3d	71.1 ± 0.9 ^d^	12.9 ± 0.2 ^e^	1670.8 ± 6.7 ^k^	167.9 ± 0.6 ^b^	157.3 ± 0.1 ^k^	38.0 ± 0.4 ^b^	19.1 ± 0.2 ^c^	4.6 ± 0.1 ^b^	8.1 ± 0.1 ^e^	5.9 ± 0.1 ^fg^
4d	71.3 ± 1.5 ^cd^	6.1 ± 0.3 ^a^	1463.7 ± 1.2 ^e^	161.6 ± 1.5 ^b^	157.8 ± 1.2 ^k^	37.6 ± 0.5 ^b^	19.1 ± 0.2 ^c^	4.7 ± 0.1 ^b^	8.7 ± 0.1 ^f^	2.3 ± 0.1 ^a^
1e	108.0 ± 1.4 ^i^	11.3 ± 0.2 ^d^	1541.6 ± 4.4 ^h^	278.3 ± 0.8 ^k^	47.6 ± 0.8 ^b^	35.1 ± 1.4 ^a^	30.8 ± 0.3 ^k^	-	9.2 ± 0.2 ^g^	5.7 ± 0.1 ^f^
2e	116.3 ± 0.2 ^k^	12.9 ± 0.1 ^e^	1708.2 ± 7.3 ^l^	261.1 ± 1.2 ^j^	104.0 ± 0.7 ^g^	46.7 ± 0.1 ^g^	26.4 ± 0.1 ^h^	-	12.1 ± 0.2 ^i^	7.5 ± 0.1 ^i^
3e	79.5 ± 0.1 ^f^	13.5 ± 0.4 ^e^	1666.0 ± 11.8 ^jk^	210.6 ± 1.5 ^f^	126.3 ± 2.0 ^h^	40.9 ± 0.2 ^cd^	22.2 ± 0.1 ^e^	-	8.5 ± 0.1 ^ef^	6.0 ± 0.1 ^g^
4e	25.4 ± 0.2 ^a^	13.3 ± 0.3 ^e^	1605.8 ± 5.9 ^i^	206.1 ± 1.1 ^f^	129.3 ± 1.4 ^hi^	37.5 ± 1.4 ^ab^	20.7 ± 0.1 ^d^	-	7.1 ± 0.1 ^d^	5.3 ± 0.1 ^e^
Sample	Methyl Dihydrojasmonate	Ethyl Myristate	Isopropyl Myristate	Diisobutyl Phthalate	Methyl Palmitate	Dibutyl Phthalate	Ethyl Palmitate	Ethyl Linoleate	Ethyl Oleate	Ethyl Stearate
Me20	1.7 ± 0.1 ^bc^	12.1 ± 0.4 ^d^	16.0 ± 0.3 ^h^	22.6 ± 0.9 ^de^	-	14.9 ± 0.3 ^b^	104.1 ± 2.2 ^d^	12.9 ± 0.1 ^f^	3.4 ± 0.1 ^de^	33.8 ± 0.8 ^l^
1a	1.1 ± 0.1 ^a^	8.6 ± 0.2 ^b^	13.4 ± 0.1 ^e^	21.6 ± 0.4 ^c^	-	16.3 ± 0.1 ^c^	114.4 ± 0.9 ^f^	11.2 ± 0.1 ^e^	3.5 ± 0.1 ^de^	47.6 ± 0.5 ^o^
2a	1.2 ± 0.1 ^a^	7.0 ± 0.2 ^a^	10.3 ± 0.1 ^b^	19.4 ± 0.1 ^b^	-	14.0 ± 0.2 ^ab^	132.6 ± 3.2 ^i^	7.2 ± 0.2 ^b^	2.0 ± 0.1 ^a^	51.1 ± 0.4 ^p^
3a	1.1 ± 0.1 ^a^	7.0 ± 0.1 ^a^	10.2 ± 0.3 ^b^	21.0 ± 0.1 ^c^	-	14.6 ± 0.4 ^b^	104.9 ± 5.9 ^de^	7.6 ± 0.2 ^b^	1.9 ± 0.1 ^a^	41.3 ± 0.5 ^n^
4a	-	7.0 ± 0.2 ^a^	10.2 ± 0.1 ^b^	16.9 ± 0.1 ^a^	-	13.1 ± 0.5 ^a^	92.1 ± 0.3 ^c^	7.4 ± 0.2 ^b^	2.1 ± 0.1 ^ab^	39.1 ± 0.1 ^m^
1b	1.1 ± 0.1 ^a^	18.8 ± 0.4 ^j^	11.9 ± 0.5 ^cd^	20.9 ± 0.4 ^c^	10.5 ± 0.1 ^g^	19.2 ± 0.5 ^d^	118.9 ± 0.7 ^g^	10.4 ± 0.1 ^d^	2.4 ± 0.1 ^b^	20.3 ± 0.3 ^f^
2b	1.9 ± 0.1 ^c^	25.9 ± 0.1 ^m^	13.7 ± 0.3 ^ef^	26.6 ± 0.2 ^g^	11.8 ± 0.2 ^h^	26.3 ± 0.1 ^g^	136.6 ± 0.4 ^i^	13.4 ± 0.1 ^g^	2.7 ± 0.1 ^c^	25.6 ± 0.5 ^i^
3b	1.8 ± 0.1 ^c^	19.8 ± 0.1 ^k^	14.3 ± 0.2 ^f^	27.9 ± 0.1 ^h^	7.5 ± 0.1 ^d^	30.8 ± 0.1 ^h^	154.6 ± 1.8 ^l^	27.9 ± 0.1 ^k^	4.7 ± 0.1 ^f^	28.9 ± 0.2 ^j^
4b	1.8 ± 0.1 ^c^	10.5 ± 0.1 ^c^	15.1 ± 0.1 ^g^	32.0 ± 0.6 ^j^	8.5 ± 0.1 ^e^	30.9 ± 0.4 ^h^	119.9 ± 0.1 ^gh^	28.8 ± 0.2 ^l^	5.1 ± 0.1 ^g^	28.7 ± 0.1 ^j^
1c	1.8 ± 0.1 ^c^	12.9 ± 0.3 ^e^	10.2 ± 0.2 ^b^	33.8 ± 0.7 ^k^	5.5 ± 0.1 ^b^	31.0 ± 0.3 ^h^	120.4 ± 0.8 ^h^	19.3 ± 0.2 ^j^	5.1 ± 0.1 ^g^	22.7 ± 0.3 ^h^
2c	2.1 ± 0.1 ^d^	13.8 ± 0.2 ^f^	14.6 ± 0.1 ^f^	41.0 ± 0.5 ^l^	8.4 ± 0.1 ^e^	36.0 ± 0.1 ^j^	148.0 ± 0.8 ^j^	35.7 ± 0.2 ^m^	5.5 ± 0.1 ^h^	30.6 ± 0.2 ^k^
3c	2.1 ± 0.1 ^d^	14.4 ± 0.3 ^g^	14.8 ± 0.2 ^fg^	33.9 ± 0.3 ^k^	8.0 ± 0.1 ^e^	32.6 ± 0.1 ^i^	151.6 ± 0.1 ^k^	19.8 ± 0.2 ^j^	3.7 ± 0.1 ^e^	23.1 ± 0.1 ^h^
4c	1.8 ± 0.1 ^c^	17.7 ± 0.1 ^i^	17.9 ± 0.2 ^i^	28.1 ± 0.2 ^h^	9.9 ± 0.2 ^fg^	26.0 ± 0.1 ^g^	158.6 ± 0.9 ^m^	19.8 ± 0.1 ^j^	3.5 ± 0.1 ^de^	21.3 ± 0.1 ^g^
1d	1.2 ± 0.1 ^a^	20.6 ± 0.3 ^l^	11.4 ± 0.1 ^c^	19.5 ± 0.3 ^b^	4.5 ± 0.1 ^a^	18.4 ± 0.2 ^d^	81.3 ± 0.8 ^b^	6.6 ± 0.1 ^a^	2.3 ± 0.1 ^b^	11.1 ± 0.1 ^d^
2d	2.1 ± 0.1 ^d^	33.8 ± 0.1 ^o^	10.1 ± 0.1 ^b^	23.8 ± 0.2 ^e^	6.1 ± 0.1 ^c^	33.5 ± 0.7 ^i^	103.5 ± 2.1 ^d^	15.6 ± 0.1 ^h^	3.2 ± 0.1 ^d^	10.5 ± 0.2 ^c^
3d	1.9 ± 0.1 ^c^	26.6 ± 0.4 ^m^	13.6 ± 0.3 ^e^	30.3 ± 0.1 ^i^	6.2 ± 0.1 ^c^	42.9 ± 0.3 ^l^	111.2 ± 1.0 ^e^	15.5 ± 0.2 ^h^	2.9 ± 0.1 ^c^	12.1 ± 0.1 ^e^
4d	1.5 ± 0.1 ^b^	26.2 ± 0.2 ^m^	14.1 ± 0.1 ^ef^	33.3 ± 0.3 ^k^	7.0 ± 0.1 ^d^	33.3 ± 0.2 ^i^	120.4 ± 0.3 ^h^	15.4 ± 0.1 ^h^	2.4 ± 0.1 ^b^	23.5 ± 0.1 ^h^
1e	1.5 ± 0.1 ^b^	15.5 ± 0.1 ^h^	8.5 ± 0.1 ^a^	23.0 ± 0.2 ^d^	5.7 ± 0.1 ^b^	20.7 ± 0.2 ^e^	93.2 ± 1.3 ^c^	13.3 ± 0.2 ^fg^	3.8 ± 0.1 ^e^	10.5 ± 0.2 ^c^
2e	1.9 ± 0.1 ^c^	30.5 ± 0.3 ^n^	12.7 ± 0.2 ^d^	23.0 ± 0.2 ^d^	9.0 ± 0.1 ^f^	39.3 ± 0.1 ^k^	109.5 ± 0.5 ^e^	18.2 ± 0.2 ^i^	2.7 ± 0.1 ^c^	11.7 ± 0.1 ^d^
3e	1.8 ± 0.1 ^c^	19.7 ± 0.1 ^k^	11.0 ± 0.1 ^c^	24.8 ± 0.1 ^f^	7.5 ± 0.1 ^d^	24.2 ± 0.1 ^f^	73.1 ± 0.3 ^a^	9.2 ± 0.1 ^c^	2.3 ± 0.1 ^b^	8.0 ± 0.1 ^a^
4e	1.6 ± 0.1 ^bc^	20.7 ± 0.1 ^l^	10.5 ± 0.1 ^b^	22.9 ± 0.1 ^d^	6.6 ± 0.1 ^c^	23.4 ± 0.3 ^f^	73.3 ± 1.2 ^a^	9.1 ± 0.1 ^c^	2.2 ± 0.1 ^ab^	9.8 ± 0.1 ^b^

Abbreviations: Me20—2020 vintage Merlot sample prior storage; a—stainless-steel tank; b—wooden barrel with excellent medium toasting; c—wooden barrel with excellent medium plus toasting; d—wooden barrel with excellent medium long toasting; e—wooden barrel with premium medium toasting1a, 1b, 1c, 1d, 1e—sampling in June 2021; 2a, 2b, 2c, 2d, 2e—sampling in September 2021; 3a–3e—sampling in December 2021; 4a, 4b, 4c, 4d, 4e—sampling in March 2022.

**Table 10:** Ester concentrations (µg/L) in aroma profile of 2021 vintage Merlot and samples obtained during 12-month storage in different vessels. Different superscript letters (a–o) in the same column indicate statistical differences determined by ANOVA and Fisher’s (LSD) test with *p* < 0.05.

Sample	Ethyl Hexanoate	Ethyl 4-hydroxybutanoate	Diethyl Succinate	Ethyl Octanoate	Ethyl Hydrogen Succinate	Phenethyl Acetate	Ethyl Decanoate	Ethyl Cinnamate	Ethyl Vanillate	Ethyl Laurate
Me21	90.4 ± 1.6 ^c^	26.4 ± 0.3 ^h^	663.4 ± 9.9 ^b^	186.6 ± 3.0 ^e^	238.5 ± 3.9 ^h^	55.0 ± 0.6 ^g^	20.5 ± 0.1 ^b^	-	2.9 ± 0.1 ^b^	5.8 ± 0.1 ^e^
1A	94.0 ± 0.8 ^d^	27.6 ± 0.1 ^i^	687.9 ± 0.9 ^c^	210.8 ± 5.1 ^gh^	244.5 ± 1.4 ^i^	66.7 ± 0.9 ^j^	21.4 ± 0.5 ^c^	-	2.3 ± 0.1 ^a^	5.6 ± 0.1 ^de^
2A	104.0 ± 2.8 ^ef^	25.5 ± 0.2 ^g^	743.5 ± 4.9 ^f^	220.0 ± 0.9 ^i^	206.9 ± 2.5 ^f^	45.1 ± 0.2 ^d^	20.6 ± 0.2 ^b^	-	2.6 ± 0.1 ^b^	5.3 ± 0.2 ^d^
3A	120.9 ± 2.1 ^h^	22.9 ± 0.1 ^e^	691.9 ± 0.3 ^d^	257.5 ± 3.4 ^k^	196.8 ± 0.8 ^d^	50.3 ± 1.2 ^e^	20.0 ± 0.2 ^b^	-	2.3 ± 0.1 ^a^	4.8 ± 0.1 ^c^
4A	100.6 ± 1.1 ^e^	15.8 ± 0.1 ^b^	583.9 ± 12.6 ^a^	233.5 ± 1.4 ^j^	195.2 ± 2.7 ^d^	50.3 ± 1.5 ^e^	20.3 ± 0.2 ^b^	-	2.0 ± 0.1 ^a^	4.3 ± 0.1 ^ab^
1B	109.0 ± 1.7 ^f^	25.6 ± 0.1 ^g^	762.7 ± 0.2 ^g^	218.4 ± 1.2 ^i^	252.8 ± 2.3 ^j^	58.5 ± 0.7 ^h^	27.5 ± 0.3 ^f^	-	3.7 ± 0.1 ^d^	4.5 ± 0.1 ^b^
2B	119.9 ± 0.8 ^h^	26.7 ± 0.1 ^h^	720.3 ± 3.4 ^e^	203.9 ± 3.1 ^g^	288.4 ± 0.8 ^m^	55.0 ± 0.3 ^g^	24.2 ± 0.1 ^d^	-	4.1 ± 0.1 ^e^	5.3 ± 0.1 ^d^
3B	120.0 ± 0.9 ^h^	28.2 ± 0.1 ^j^	720.6 ± 3.9 ^e^	203.0 ± 2.8 ^g^	207.8 ± 4.4 ^f^	60.5 ± 0.1 ^i^	20.3 ± 0.3 ^b^	-	3.7 ± 0.1 ^d^	4.2 ± 0.1 ^a^
4B	106.6 ± 0.7 ^f^	32.5 ± 0.2 ^k^	1054.1 ± 4.4 ^m^	194.1 ± 2.3 ^f^	195.7 ± 2.5 ^d^	55.8 ± 0.1 ^g^	24.8 ± 0.1 ^d^	-	3.2 ± 0.1 ^bc^	4.2 ± 0.1 ^a^
1C	102.6 ± 0.3 ^e^	12.5 ± 0.1 ^a^	910.5 ± 15.8 ^l^	223.3 ± 3.3 ^i^	266.2 ± 3.8 ^k^	67.1 ± 0.8 ^j^	27.7 ± 0.3 ^f^	-	3.2 ± 0.1 ^bc^	10.6 ± 0.1 ^k^
2C	87.2 ± 0.4 ^b^	12.8 ± 0.1 ^a^	1145.5 ± 6.0 ^n^	251.4 ± 3.9 ^k^	290.1 ± 3.9 ^m^	80.9 ± 0.7 ^k^	22.1 ± 0.1 ^c^	-	4.9 ± 0.1 ^g^	8.3 ± 0.1 ^i^
3C	93.2 ± 1.2 ^cd^	12.5 ± 0.4 ^a^	875.1 ± 9.0 ^jk^	231.5 ± 0.9 ^j^	217.4 ± 0.4 ^g^	57.4 ± 0.4 ^h^	19.0 ± 0.1 ^a^	-	3.4 ± 0.1 ^c^	8.4 ± 0.1 ^i^
4C	109.7 ± 0.1 ^f^	12.4 ± 0.1 ^a^	873.5 ± 1.8 ^j^	234.4 ± 0.1 ^j^	200.9 ± 0.6 ^e^	55.1 ± 3.1 ^fgh^	20.9 ± 0.1 ^bc^	-	3.2 ± 0.1 ^bc^	6.5 ± 0.1 ^g^
1D	93.5 ± 0.1 ^d^	22.4 ± 0.2 ^d^	810.3 ± 8.1 ^hi^	122.0 ± 1.8 ^a^	263.1 ± 1.7 ^k^	44.1 ± 0.8 ^d^	27.7 ± 0.3 ^f^	-	5.2 ± 0.1 ^gh^	4.5 ± 0.1 ^b^
2D	108.0 ± 1.7 ^f^	22.1 ± 0.3 ^d^	889.3 ± 4.2 ^k^	211.9 ± 2.8 ^h^	280.2 ± 2.3 ^l^	39.0 ± 0.5 ^b^	22.1 ± 0.1 ^c^	-	5.4 ± 0.1 ^h^	9.9 ± 0.1 ^j^
3D	87.2 ± 0.2 ^b^	16.2 ± 0.1 ^c^	906.3 ± 1.4 ^l^	168.4 ± 1.1 ^d^	233.6 ± 1.1 ^h^	42.3 ± 0.6 ^c^	25.1 ± 0.1 ^e^	-	4.5 ± 0.1 ^f^	7.5 ± 0.1 ^h^
4D	87.6 ± 0.5 ^b^	16.2 ± 0.2 ^c^	904.3 ± 2.5 ^l^	153.9 ± 1.0 ^c^	233.8 ± 1.4 ^h^	42.2 ± 0.3 ^c^	25.0 ± 0.1 ^e^	-	9.3 ± 0.1 ^i^	6.1 ± 0.1 ^f^
1E	94.7 ± 1.7 ^d^	21.8 ± 0.2 ^d^	827.8 ± 8.8 ^i^	205.1 ± 0.4 ^g^	109.7 ± 1.0 ^a^	52.6 ± 0.5 ^f^	24.3 ± 0.1 ^d^	-	3.2 ± 0.1 ^bc^	4.5 ± 0.1 ^b^
2E	116.8 ± 0.7 ^g^	22.5 ± 0.4 ^de^	1015.4 ± 12.1 ^m^	198.3 ± 2.3 ^f^	150.1 ± 0.4 ^b^	60.7 ± 1.3 ^i^	24.5 ± 0.2 ^d^	-	4.5 ± 0.1 ^f^	4.2 ± 0.1 ^a^
3E	87.5 ± 0.1 ^b^	23.9 ± 0.1 ^f^	833.5 ± 17.7 ^i^	159.3 ± 2.0 ^c^	158.4 ± 1.4 ^c^	38.7 ± 0.2 ^b^	21.3 ± 0.1 ^c^	-	2.2 ± 0.1 ^a^	4.0 ± 0.1 ^a^
4E	76.4 ± 0.7 ^a^	23.5 ± 0.4 ^ef^	796.7 ± 11.2 ^h^	134.4 ± 3.6 ^b^	157.5 ± 1.0 ^c^	33.9 ± 0.4 ^a^	18.5 ± 0.1 ^a^	-	2.2 ± 0.1 ^a^	4.3 ± 0.2 ^ab^
Sample	Methyl Dihydrojasmonate	Ethyl Myristate	Isopropyl Myristate	Diisobutyl Phthalate	Methyl Palmitate	Dibutyl Phthalate	Ethyl Palmitate	Ethyl Linoleate	Ethyl Oleate	Ethyl Stearate
Me21	4.8 ± 0.1 ^h^	25.0 ± 0.1 ^d^	17.4 ± 0.2 ^e^	33.2 ± 0.2 ^g^	7.3 ± 0.1 ^e^	24.4 ± 0.4 ^c^	106.0 ± 1.1 ^f^	18.6 ± 0.1 ^f^	6.0 ± 0.1 ^f^	17.6 ± 0.2 ^fg^
1A	3.5 ± 0.1 ^ef^	18.2 ± 0.4 ^b^	15.8 ± 0.2 ^c^	34.1 ± 0.4 ^gh^	6.1 ± 0.1 ^d^	27.1 ± 0.4 ^e^	119.3 ± 1.1 ^h^	15.3 ± 0.1 ^c^	6.8 ± 0.1 ^g^	20.3 ± 0.3 ^h^
2A	3.3 ± 0.1 ^e^	15.9 ± 0.2 ^a^	12.3 ± 0.1 ^a^	31.1 ± 0.3 ^f^	4.4 ± 0.1 ^c^	25.3 ± 0.1 ^d^	131.8 ± 0.2 ^j^	13.3 ± 0.1 ^b^	6.0 ± 0.1 ^f^	21.7 ± 0.5 ^i^
3A	3.6 ± 0.1 ^f^	15.8 ± 0.1 ^a^	12.2 ± 0.1 ^a^	36.0 ± 0.8 ^i^	2.1 ± 0.1 ^a^	25.2 ± 0.3 ^cd^	93.7 ± 0.4 ^d^	15.5 ± 0.1 ^c^	4.1 ± 0.1 ^b^	20.7 ± 0.2 ^h^
4A	3.4 ± 0.1 ^ef^	15.0 ± 0.1 ^a^	12.1 ± 0.1 ^a^	30.6 ± 0.3 ^ef^	2.1 ± 0.1 ^a^	18.3 ± 0.1 ^a^	79.3 ± 0.2 ^b^	15.1 ± 0.1 ^c^	4.7 ± 0.1 ^c^	17.7 ± 0.4 ^fg^
1B	3.7 ± 0.1 ^f^	31.0 ± 0.3 ^f^	15.8 ± 0.1 ^c^	30.8 ± 0.1 ^ef^	9.0 ± 0.1 ^g^	36.4 ± 0.2 ^i^	114.3 ± 1.9 ^g^	13.1 ± 0.1 ^b^	4.9 ± 0.1 ^cd^	15.3 ± 0.3 ^d^
2B	4.1 ± 0.1 ^g^	34.2 ± 0.3 ^h^	20.4 ± 0.5 ^g^	34.7 ± 0.3 ^h^	10.4 ± 0.2 ^h^	41.4 ± 0.1 ^k^	120.0 ± 0.2 ^h^	16.5 ± 0.1 ^d^	5.6 ± 0.1 ^e^	17.0 ± 0.1 ^f^
3B	4.1 ± 0.1 ^g^	27.1 ± 0.3 ^e^	23.4 ± 0.1 ^h^	37.5 ± 0.2 ^j^	8.8 ± 0.1 ^fg^	42.7 ± 0.2 ^l^	127.4 ± 2.7 ^i^	17.9 ± 0.1 ^e^	6.1 ± 0.1 ^f^	17.9 ± 0.1 ^fg^
4B	4.0 ± 0.1 ^g^	19.3 ± 0.1 ^c^	23.5 ± 0.3 ^h^	41.9 ± 0.4 ^l^	9.5 ± 0.1 ^g^	42.6 ± 0.1 ^l^	115.6 ± 0.4 ^g^	18.7 ± 0.1 ^f^	7.3 ± 0.1 ^h^	17.7 ± 0.3 ^fg^
1C	4.9 ± 0.1 ^h^	37.4 ± 0.3 ^i^	16.7 ± 0.2 ^d^	41.1 ± 0.4 ^l^	9.6 ± 0.1 ^g^	34.9 ± 0.2 ^h^	140.1 ± 1.4 ^l^	26.0 ± 0.1 ^h^	6.2 ± 0.1 ^f^	16.5 ± 0.1 ^e^
2C	6.2 ± 0.1 ^k^	48.7 ± 0.2 ^l^	17.7 ± 0.2 ^e^	63.2 ± 0.9 ^m^	10.6 ± 0.2 ^h^	34.8 ± 0.1 ^h^	145.5 ± 0.1 ^m^	32.3 ± 0.3 ^j^	11.4 ± 0.1 ^j^	17.9 ± 0.1 ^fg^
3C	5.8 ± 0.1 ^j^	44.5 ± 0.2 ^j^	18.8 ± 0.1 ^f^	39.9 ± 0.8 ^k^	13.3 ± 0.3 ^i^	33.1 ± 0.2 ^g^	152.5 ± 0.4 ^n^	29.6 ± 0.1 ^i^	10.5 ± 0.1 ^i^	17.8 ± 0.1 ^fg^
4C	5.3 ± 0.1 ^i^	46.8 ± 0.2 ^k^	20.1 ± 0.1 ^g^	36.9 ± 0.3 ^ij^	15.4 ± 0.2 ^j^	30.9 ± 0.4 ^g^	164.3 ± 1.1 ^o^	29.7 ± 0.1 ^i^	10.4 ± 0.1 ^i^	16.2 ± 0.1 ^e^
1D	1.9 ± 0.1 ^b^	26.7 ± 0.4 ^e^	13.6 ± 0.3 ^b^	13.7 ± 0.1 ^a^	7.8 ± 0.1 ^e^	25.3 ± 0.1 ^d^	81.6 ± 0.5 ^c^	11.8 ± 0.1 ^a^	4.9 ± 0.1 ^cd^	11.1 ± 0.3 ^b^
2D	3.9 ± 0.1 ^fg^	38.0 ± 0.9 ^i^	17.9 ± 0.1 ^e^	29.2 ± 0.1 ^d^	8.5 ± 0.1 ^f^	36.7 ± 0.2 ^ij^	93.0 ± 2.7 ^d^	18.0 ± 0.2 ^ef^	5.2 ± 0.1 ^d^	10.1 ± 0.1 ^a^
3D	2.3 ± 0.1 ^cd^	31.8 ± 0.7 ^f^	19.7 ± 0.1 ^g^	30.4 ± 0.2 ^e^	6.5 ± 0.1 ^d^	38.4 ± 0.8 ^h^	127.0 ± 0.2 ^i^	18.8 ± 0.6 ^f^	3.2 ± 0.1 ^a^	14.1 ± 0.2 ^c^
4D	2.2 ± 0.1 ^c^	32.4 ± 0.4 ^g^	24.1 ± 0.3 ^i^	33.7 ± 0.5 ^g^	8.9 ± 0.1 ^fg^	37.2 ± 0.1 ^j^	136.2 ± 0.1 ^k^	18.3 ± 0.3 ^ef^	3.2 ± 0.1 ^a^	16.1 ± 0.3 ^e^
1E	2.5 ± 0.1 ^d^	26.6 ± 0.5 ^e^	16.5 ± 0.1 ^d^	29.1 ± 0.2 ^d^	7.8 ± 0.1 ^e^	29.1 ± 0.4 ^f^	100.5 ± 0.7 ^e^	18.6 ± 0.3 ^f^	10.9 ± 0.1 ^i^	14.5 ± 0.1 ^c^
2E	2.6 ± 0.1 ^d^	33.7 ± 0.3 ^h^	18.3 ± 0.1 ^g^	24.1 ± 0.5 ^c^	4.4 ± 0.1 ^c^	28.2 ± 0.2 ^f^	114.1 ± 1.3 ^g^	21.6 ± 0.2 ^g^	7.6 ± 0.1 ^h^	18.0 ± 0.1 ^g^
3E	1.8 ± 0.1 ^b^	26.7 ± 0.1 ^e^	16.1 ± 0.2 ^cd^	23.5 ± 0.1 ^a^	3.3 ± 0.1 ^b^	24.8 ± 0.4 ^cd^	60.0 ± 0.6 ^a^	15.5 ± 0.1 ^c^	6.7 ± 0.1 ^g^	9.9 ± 0.1 ^a^
4E	1.4 ± 0.1 ^a^	26.8 ± 0.4 ^e^	16.1 ± 0.4 ^cd^	21.5 ± 0.1 ^b^	2.0 ± 0.1 ^a^	21.1 ± 0.3 ^b^	60.3 ± 0.3 ^a^	15.7 ± 0.1 ^c^	6.6 ± 0.1 ^g^	10.2 ± 0.3 ^a^

Abbreviations: Me21—2021 vintage Merlot sample prior storage; A—stainless-steel tank; B—wooden barrel with excellent medium toasting; C—wooden barrel with excellent medium plus toasting; D—wooden barrel with excellent medium long toasting; E—wooden barrel with premium medium toasting; 1A–1E—sampling in August 2022; 2A–2E—sampling in November 2022; 3A–3E—sampling in February 2023; 4A–4E—sampling in May 2023.

**Table 11:** The initial and highest concentrations after 12-month storage of most abundant and marked aroma compounds in 2020 and 2021 vintage Merlot and samples obtained from different storage vessels.

Compound	Initial Concentration		Highest Concentration *		Description
	Me20	Me21	Me20	Me21	
Acetic acid	631.0 μg/L	609.6 μg/L	780. 5 μg/L (EMT+)	732.9 μg/L (EMT+)	Most abundant acid, contributing to the light vinegar aroma and freshness
4-ethylphenol	47.0 μg/L	-	62.8 μg/L (EMT)	55.5 μg/L (PMT)	Smoky aroma, usually formed during storage in wooden barrel
4-ethylguaiacol	13.2 μg/L	-	17.3 μg/L (EMT+)	12.3 μg/L (PMT)	Smoky aroma, usually formed during storage in wooden barrel
4-propylguaiacol	-	-	4.7 μg/L (EMT)	-	Spicy aroma, usually formed during storage in wooden barrel
Isoamyl alcohol	10 mg/L	6.5 mg/L	16.4 mg/L (EMLT)	16.7 mg/L (EMLT)	Most abundant alcohol, fruity aroma
2-phenylethanol	3.1 mg/L	2.5 mg/L	4.1 mg/L (EMT)	3.2 mg/L (EMT)	Second most abundant alcohol, floral aroma
Lily aldehyde	-	7.1 μg/L	9.1 μg/L (EMT+)	23.3 μg/L (EMT+)	Floral aroma, formed or increased during storage
Eugenol	-	-	11.1 μg/L (PMT)	4.6 μg/L (EMT+)	Aroma of cloves, spicy notes, usually formed during storage in wooden barrel
Diethyl succinate	1039.4 μg/L	663.4 μg/L	1639.0 μg/L (EMT)	1054.1 μg/L (EMT)	Most abundant ester, fruity aroma
Ethyl vanillate	6.1 μg/L	2.9 μg/L	8.9 μg/L (EMT+)	9.3 μg/L (EMLT)	Smoky aroma, usually present in red wine from wooden barrels

* The highest concentration measured after 12 months of storage. Abbreviations: Me20—initial 2020 vintage Merlot red wine; Me21—initial 2021 vintage Merlot red wine; EMT—Excellence oak barrel with medium toasting; EMT+—Excellence oak barrel with medium plus toasting; EMLT—Excellence oak barrel with medium long toasting; PMT—Premium oak barrel with medium toasting.

**Table 12:** Organoleptic evaluation of 2020 vintage Merlot and samples obtained during 12-month storage in different vessels by 100-point scale.

Parameter	Me20	1a	2a	3a	4a	1b	2b	3b	4b	1c	2c	3c	4c	1d	2d	3d	4d	1e	2e	3e	4e
Visual	15.0	15.0	15.0	15.0	15.0	15.0	15.0	15.0	15.0	15.0	15.0	15.0	15.0	15.0	15.0	15.0	15.0	15.0	15.0	15.0	15.0
Nose	26.3	25.3	27.0	23.7	22.7	29.7	28.0	26.0	23.3	25.0	26.7	26.0	26.0	25.7	23.3	23.0	21.7	27.7	27.0	27.3	26.3
Taste	37.7	37.3	37.3	37.3	35.7	40.3	40.0	41.0	37.7	37.3	35.7	35.7	38.3	36.0	34.0	37.0	33.3	38.7	38.7	39.7	40.0
Harmony	10.0	9.7	9.3	9.7	9.7	10.0	9.7	10.7	10.0	9.7	9.7	9.3	9.7	9.0	9.3	9.3	9.7	10.0	10.0	9.7	10.0
Total	89.0	87.3	88.7	85.7	83.0	95.0	92.7	92.7	86.0	87.0	87.0	86.0	89.0	85.7	81.7	84.3	79.7	91.3	90.7	91.7	91.3

Points were expressed as the average value of three different judges. Maximal points for visual (clarity and colour) was 15, for nose (aroma purity, intensity and quality) was 30, for taste (purity, intensity, persistence and quality) was 44 and for harmony was 11, making a total of 100 points.

**Table 13:** Organoleptic evaluation of 2021 vintage Merlot and samples obtained during 12-month storage in different vessels by 100-point scale.

Parameter	Me21	1A	2A	3A	4A	1B	2B	3B	4B	1C	2C	3C	4C	1D	2D	3D	4D	1E	2E	3E	4E
Visual	15.0	15.0	15.0	15.0	15.0	15.0	15.0	15.0	15.0	15.0	15.0	15.0	15.0	15.0	15.0	15.0	15.0	15.0	15.0	15.0	15.0
Nose	24.0	23.7	23.0	24.3	25.3	26.0	25.7	26.7	28.0	25.0	25.3	25.0	24.0	25.3	22.3	25.0	25.3	24.0	27.3	27.7	28.3
Taste	34.7	34.3	36.3	36.3	39.0	35.0	38.7	40.7	40.0	34.0	41.3	37.3	37.0	34.7	39.7	36.7	36.3	37.3	42.7	39.7	42.3
Harmony	10.0	9.3	9.7	10.0	10.0	10.0	10.7	10.0	10.0	9.3	10.7	9.7	10.0	9.3	10.0	9.7	10.0	10.0	10.0	9.3	10.0
Total	83.7	82.3	84.0	85.7	89.3	86.0	90.0	92.3	93.0	83.3	92.3	87.0	86.0	84.3	87.0	86.3	86.7	86.3	95.0	91.7	95.7

Points were expressed as the average value of three different judges. Maximal points for visual (clarity and colour) was 15, for nose (aroma purity, intensity and quality) was 30, for taste (purity, intensity, persistence and quality) was 44 and for harmony was 11, making a total of 100 points.

## Data Availability

Data are contained within the article.
